# Strain release of substoichiometric (Zr,Y)O$_{2-x}$ phases formed by electrochemical reduction in single crystalline YSZ

**DOI:** 10.1038/s41598-026-45838-x

**Published:** 2026-04-11

**Authors:** Christian Rodenbücher, Dominik Wrana, Benedykt R. Jany, Grzegorz Cempura, Paulina Indyka, Adam Kruk, Kristof Szot, Franciszek Krok, Carsten Korte

**Affiliations:** 1 Institute of Energy Technologies (IET-4), Forschungszentrum Jülich GmbH, Jülich, 52425 Germany; 2 Marian Smoluchowski Institute of Physics, Jagiellonian University, Kraków, 30-348 Poland; 3 AGH University of Krakow, Kraków, 30-059 Poland; 4 SOLARIS National Synchrotron Radiation Centre, Jagiellonian University, Kraków, 30-392 Poland; 5 Institute of Physics, University of Silesia, Chorzów, 41-500 Poland

**Keywords:** Electrochemical reduction, Yttria stabilised zirconium dioxide, Substoichiometric ziconium-yttrium oxide, Strain release, Metastable phases, STEM/EDX study, Chemistry, Materials science

## Abstract

The formation of substoichiometric mixed zirconium-yttrium oxides by electroreduction of cubic 9.5YSZ is investigated. A strongly oxygen depleted phase with the stoichiometry (Zr,Y)$_{8.6}$O is observed, forming belt-shaped features below the sample surface in the heavily reduced region close to the cathode. It is embedded in another oxygen depleted phase with the stoichiometry (Zr,Y)$_2$O. The electroreduction is performed by drawing a DC current through a single crystal with circular platinum electrodes. The new phases, which are possibly metastable and not yet reported in literature, are identified by STEM investigations and EDX, which is used to measure the composition. The found composition indicates Zr and Y with an oxidation state of +I, respectively, between +I and 0. The (Zr,Y)$_{8.6}$O phase has a significant distortion of the original cubic symmetry. The (Zr,Y)$_2$O phase exhibits a slight, the (Zr,Y)$_{8.6}$O phase a strong decrease of the molar volume compared to unreduced YSZ. A chequerboard-like structure on the surface of the single crystal can be most probably explained by strain relaxation due to dislocation gliding. Misfit dislocations can be found in the interface between the substoichiometric phases. The induced strain due to the volume contraction may also be responsible for the known deterioration of the mechanical properties after reduction.

## Introduction

Functional oxide ceramics are often exhibited to elevated temperatures, but in various applications also to electric fields of different magnitude. The electrolyte layers in solid oxide fuel cells (SOFC) or solid oxide electrolyser cells (SOEC) are exposed to a field of about 600 V cm$^{-1}$^1,2^, the dielectric oxides in multilayer capacitors to a field of 7 kV cm$^{-1}$ and the dielectric gate oxides in field effect transistors (MOSFET) to a field up to 3 MV cm$^{-1}$^3^. Depending on the materials properties, this may induce electronic, respectively ionic currents, that can cause degradation processes. On the other hand, the occurrence of transient redox phenomena are key features of techniques like “spark plasma sintering” or “field assisted sintering” for materials synthesis (SPS/FAST)^4–7^.

The electro-colouration or blackening effect of yttria-stabilised zirconia, has been investigated extensively in the last 50 years since its first observations during the development of MHD (magnetohydrodynamic) devices^8^. Different experimental techniques including high-resolution electron microscopy^9,10^, X-ray photoelectron spectroscopy (XPS), X-ray diffraction (XRD), electron spin resonance (ESR)^11–19^, measurements of elastic properties^20^, dielectric properties^21^, electric conductivity^22–26^ and optical spectroscopy^18,26–29^ have been used to reveal the colouration process and the nature of the blackened phase.

The blackening is related to a local reduction of the material, which is electrochemically induced by lowering the oxygen activity. Alternatively, a blackening, respectively a colouration, can also be observed in the case of a chemical reduction with metallic zirconium or equilibration in an inert gas at elevated temperatures^22^, by a treatment with laser radiation^30^, X-rays^16,17,19^ or by sputtering of the surface^10^. It appears that this phenomenon is a complex multistep process and depends subtly on the local electrochemical conditions^31–40^. The detailed nature of the restructuring and the chemical composition of the (stabilised) zirconia under electrochemical polarization is still not fully understood. Depending on the current density, the reduced material undergoes a strong change in its physical properties. The electronic conductivity is highly increased and the mechanic properties degrade which restricts its use as solid electrolyte or refractory material.

In this study we investigate the (electro-)reduction of yttria-stabilised cubic zirconium dioxide to identify the oxygen deficiency phase and its structure. The reduced material is characterised by light microscopy and scanning electron microscopy to identify morphological changes on the surface and by transmission electron microscopy to identify new phases in the bulk.

## Formal considerations

### Phase diagram of zirconium and oxygen

In the literature, many studies can be found concerning the quasi-binary phase diagrams of zirconium dioxide and oxides with di- or trivalent cations able to stabilise the cubic high-temperature fluorite structure. An addition of more than 8 mol% Y$_2$O$_3$ results in a fully stabilised material without a phase transition to the tetragonal or monoclinic phase below 1000 $^{\circ }$C (8YSZ)^41^.

**Fig. 1:**
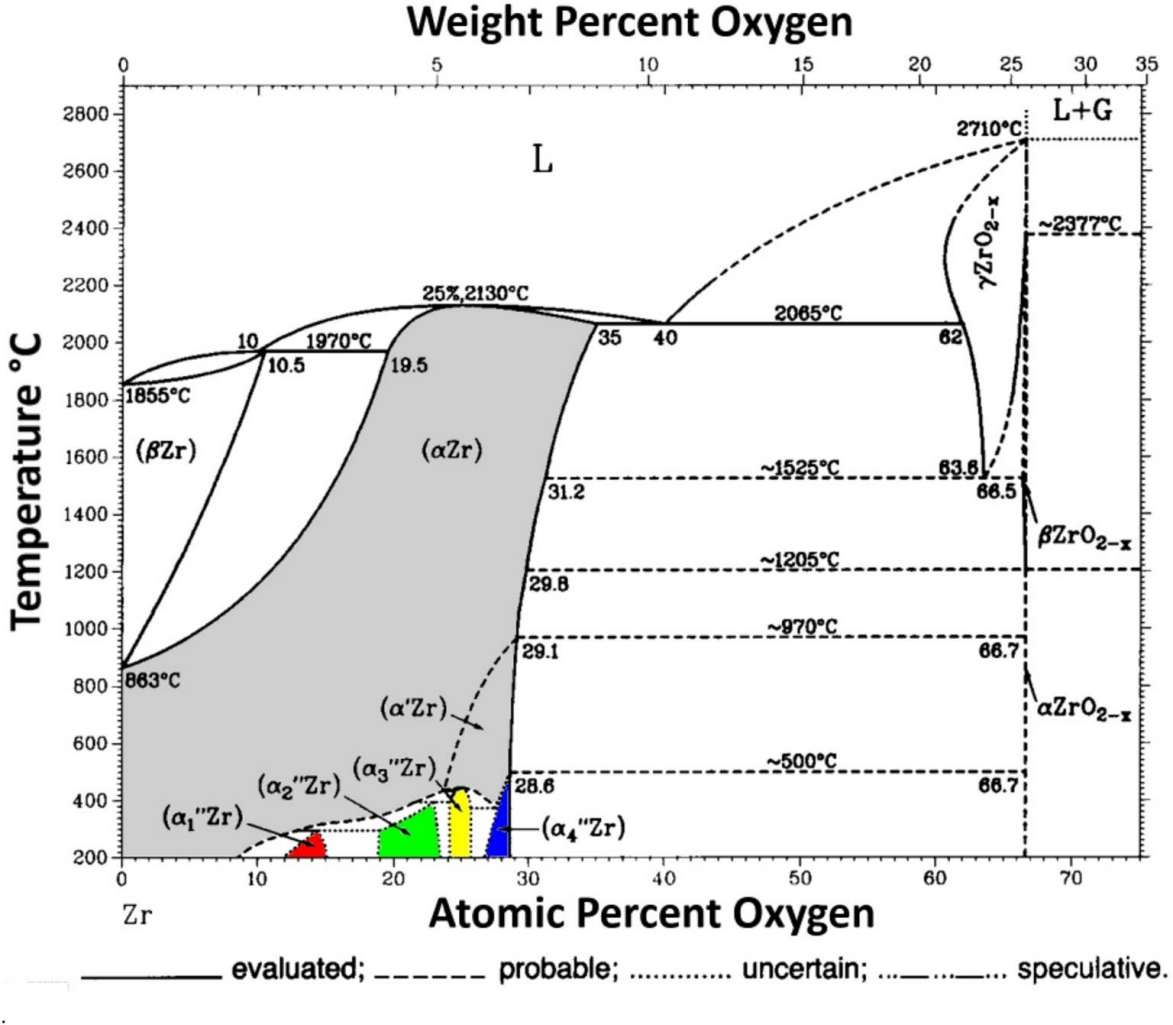
Phase diagram of the system Zr–O, adapted with permission from Abriata et al.^42^. Copyright 1986, Springer Nature. The sub-stoichiometric fully disordered and only partially ordered phases ($\alpha$Zr) and ($\alpha ^\prime$Zr) are marked in grey. The ordered intercalation phases are marked in red ($\alpha _1^{\prime \prime }$Zr), green ($\alpha _2^{\prime \prime }$Zr), yellow ($\alpha _3^{\prime \prime }$Zr) and blue ($\alpha _4^{\prime \prime }$Zr).

Regarding the binary phase diagram of zirconium and oxygen, the number of studies in the literature is comparably sparse, especially in the case of zirconium rich phases. According to Abriata et al., the stoichiometry range of the monoclinic $\alpha$ZrO$_{2-\delta }$ (P2$_1$c) phase is only small, restricting the scope to temperatures below 800 $^{\circ }$C^42^ The maximum oxygen deficiency $\delta$ is less than 10$^{-1}$. The only reported sub-stoichiometric phases are intercalation compounds based on the closed packed hexagonal structure (P6$_3$/mmc) of metallic $\alpha$-zirconium ($\alpha$Zr) with oxygen atoms randomly distributed on interstitial sites^43,44^. Depending on the oxygen fraction, ordering can appear resulting in trigonal or hexagonal structures, *e.g.*, ($\alpha _1^{\prime \prime }$Zr) with a composition of Zr$_{8}$O to Zr$_{5.6}$O, ($\alpha _2^{\prime \prime }$Zr) with a composition of Zr$_{4.5}$O to Zr$_{3.4}$O or ($\alpha _3^{\prime \prime }$Zr) with a composition of Zr$_{3.1}$O to Zr$_{2.9}$O, see Fig. 1 and Table 1^44–49^. There are relative broad ranges of stoichiometry. No ordered intercalation phases, respectively, equilibrium phases with a Zr fraction lower than Zr$_{2.5}$O can be found^42,48,50^.

**Fig. 2:**
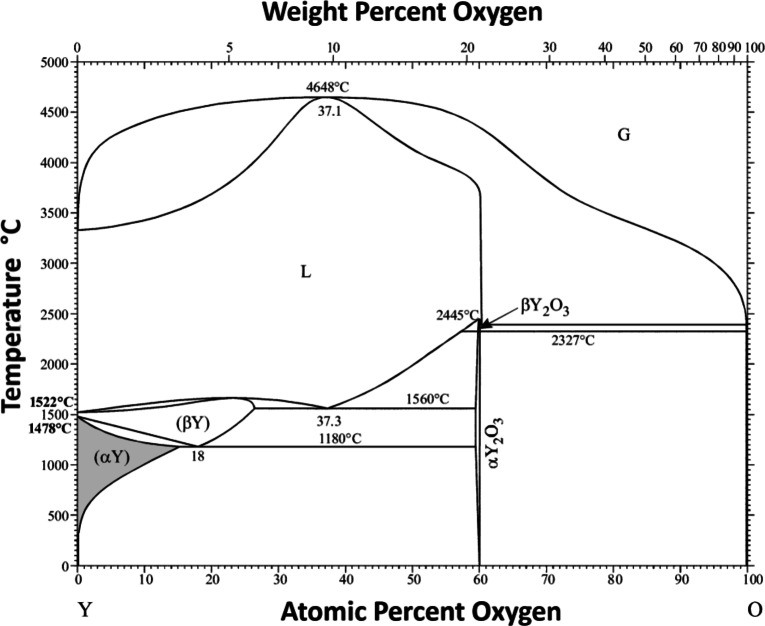
Phase diagram of the system Y – O, adapted with permission from Okamoto^51^. Copyright 2011, Springer Nature. The sub-stoichiometric disordered phase ($\alpha$Y) is marked in grey.

When performing a chemical or an electrochemical reduction, preferentially at low temperatures, sub-stoichiometric non-equilibrium (metastable) phases may also be taken into account. The Zr$_x$O phases with a Zr content lower than $x~<$ 2.5, reported in the literature, are generally not based on the closed packed hexagonal structure of metallic zirconium. Two cubic phases with the composition Zr$_2$O and ZrO were reported by Khitrova and Klechkovskaya^52^. Using electron diffraction (TEM/SAED) for analysis, they found a cubic cuprite structure (Pn$\bar{3}$m) for Zr$_2$O and a defective fluorite structure (Fm$\bar{3}$m) for ZrO. The oxygen sites are only half occupied in a random manner. Both structures were also reported in a study by Henning et al. investigating ZrO$_2$ thin films reduced in vacuum by argon sputtering^10^. Only the ZrO phase is described by Campos Neto et al. in a melt-spinning study on the system Zr–Ni–Cu, but a significantly lower lattice constant is measured^53^. However, a ZrO phase with cubic NaCl structure (Fm$\bar{3}$m) is reported by Schönberg and a Zr$_2$O phase with hexagonal superstructure based on ($\alpha$Zr) is reported by Steeb and Riekert, see Table 1^54,55^. The data in this Table is collected from the literature and is mainly based on XRD results. Only the phase diagram of the system zirconium - oxygen is available in the literature. The only thermodynamically stable phases in the system zirconium - oxygen are monoclinic ZrO$_2$ and hexagonal/trigonal Zr-rich O-intercalation compounds. Thus, the phases with a composition ZrO and Zr$_2$O are definitely metastable but reported several times in literature and included in the ICSD database. Hence, it can be assumed that also the reported metastable phases are sufficiently “stable” for diffractional analysis.

**Table 1:** Experimentally reported stable and metastable substoichiometric phases with an approximate composition of ZrO, Zr$_2$O, Zr$_3$O, and Zr$_6$O and their structural data in the binary system Zr–O. The structural data of 9.5YSZ, $\alpha$ZrO$_2$ and $\alpha$Zr is given for comparison. Campos Neto et al.^53^ refer to Khitrova et al.^52^, despite the fact of the different lattice constant.

Stoichiometry	Spacegroup	Lattice parameter	Ref.
9.5YSZ	Fm$\bar{3}$m (CaF$_2$)	*a* = 5.143 Å	“Supplemental material”, 5)
$\alpha$ZrO$_2$	P2$_1$c (Baddeleyite)	*a* = 5.105 Å, *b* = 5.208 Å, *c* = 5.254 Å $\beta$ = 98.868 $^{\circ }$	^56^
ZrO	cubic (?)	*a* = 4.600 Å	^55,57^
ZrO	Fm$\bar{3}$m (NaCl)	*a* = 4.602 Å	^54^, ICSD 76019
ZrO	Fm$\bar{3}$m (defective CaF$_2$)	*a* = 4.62 Å	^53^, ICSD 77713 (?)
ZrO	Fm$\bar{3}$m (defective CaF$_2$)	*a* = 5.11 Å	^10,52^, ICSD 77713
Zr$_2$O	Pn$\bar{3}$m (Cuprite)	*a* = 5.088 Å	^10,52^, ICSD 77714
Zr$_2$O	hexagonal (?)	*a* = 16.160 Å, *c* = 5.148 Å	^55^
Zr$_{2.98}$O	P6$_3$/mmc	*a* = 3.2510 Å, *c* = 5.1937 Å	^44^, ICSD 647685
Zr$_3$O	R32H	*a* = 5.5630 Å, *c* = 31.185 Å	^47^, ICSD 23402
Zr$_3$O	R$\bar{3}$cH	*a* = 5.6295 Å, *c* = 15.5925 Å	^46^, ICSD 27023
Zr$_3$O	P6$_3$22	*a* = 5.6172 Å, *c* = 5.1850 Å	^49^, ICSD 88316
Zr$_3$O	P6$_3$22	*a* = 5.627 Å, *c* = 5.193 Å	^45^, ICSD 42985
Zr$_3$O	P6$_3$22	*a* = 5.6295 Å, *c* = 5.1975 Å	^44^, ICSD 77715
Zr$_3$O	P6$_3$22	*a* = 5.6308 Å, *c* = 5.1898 Å	^49^, ICSD 88320
Zr$_{3.7}$O	P3c1	*a* = 5.634 Å, *c* = 15.543 Å	^45^, ICSD 42986
Zr$_{5.6}$O	P3c1	*a* = 5.624 Å, *c* = 15.510 Å	^45^
Zr$_{41.67}$O	P6$_3$mmc	*a* = 3.2333 Å, *c* = 5.1513 Å	^43^, ICSD 647697
Zr	P6$_3$mmc	*a* = 3.2340 Å, *c* = 5.1140 Å	^58^, ICSD 653528

There are also theoretical studies using DFT calculations to evaluate the stability of possible sub-stoichiometric phases. According to Burton et al. there should be a trigonal Zr$_6$O phase (R$\bar{3}$), a trigonal Zr$_3$O phase (R$\bar{3}$c), a hexagonal Zr$_3$O phase (P6$_3$22), a trigonal Zr$_{12}$O$_5$ phase (R$\bar{3}$) and a trigonal Zr$_2$O phase (P$\bar{3}$1m)^59^. The Zr$_6$O phase (R$\bar{3}$), the Zr$_3$O phase (R$\bar{3}$c) and the Zr$_2$O phase (P$\bar{3}$1m) is also reported by Zhang et al.^60^. In addition, they found a ZrO phase with hexagonal structure (P$\bar{6}$2m). According to this study, the experimentally reported cuprite-type Zr$_2$O phase should be unstable.

Regarding the binary phase diagram of yttrium and oxygen, there is not such a variety of sub-stoichiometric phases. According to Okamoto, there is only the $\alpha$Y$_2$O$_3$ phase with a cubic bixbyite structure (Ia$\bar{3}$) and an intercalation phase based on hexagonal structure (P6$_3$/mmc) of metallic $\alpha$-yttrium ($\alpha$Y) with oxygen on interstitial sites at temperatures below 800 $^{\circ }$C, see Fig. 2^51^. No other equilibrium phases in between are reported^61^. In addition, a high-pressure rock-salt type YO phase (Fm$\bar{3}$m) exists, metastable at ambient conditions^62,63^. However, regarding the ternary diagram of zirconium, yttrium and oxygen, no comparable studies can be found.

### Oxygen ion and electronic conductivity

A redox process that results in the formation of a second phase (product phase) in an oxide can take place, if the chemical potential of oxygen, *i.e.*, its partial pressure, exceeds limiting minimal or maximal values. Considering an electrochemical cell, if ionic and electronic charge transport is present, the limiting values can be reached in the vicinity of electrode/electrolyte interfaces or phase boundaries between different conductors, where the transport properties regarding ionic and electronic partial conduction are changing. Thus, for a general analysis of interface redox phenomena, it is necessary to know the partial conductivities of the present phases, respectively, the ionic and electronic transference numbers.

**Fig. 3:**
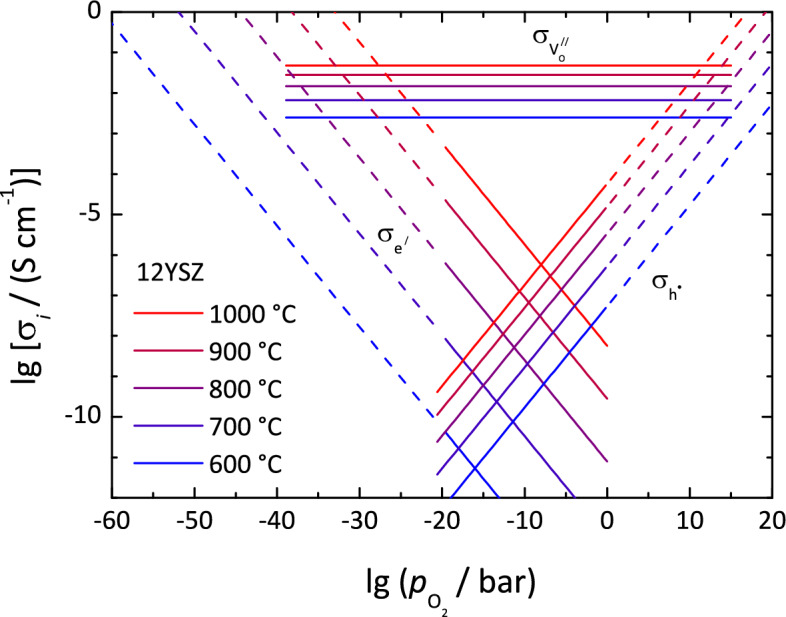
Electronic and ionic partial conductivities of 12YSZ. The oxide ion partial conductivity $\sigma _{\textrm{O}^{2-}}$ is identical to the oxide vacancy conductivity $\sigma _{\textrm{V}_\textrm{O}^{\prime \prime }}$, the electronic partial conductivity $\sigma _{\textrm{e}^-}$ is the sum of the electron and hole conductivities, $\sigma _{\textrm{e}^\prime } + \sigma _{\textrm{h}^\cdot }$. Data from Park and Blumenthal and Lomonova et al.^64,65^ The dashed regions are extrapolated.

Data for the O$^{2-}$ partial conductivity $\sigma _{\textrm{O}^{2-}}$ of unreduced YSZ is readily available for a wide range of temperatures, *e.g.*, Lomonova et al. or Zhang et al.^65,66^. Data for electronic partial conductivity $\sigma _{\textrm{e}^-}$ is comparable sparse, *e.g.*, Park and Blumenthal or Zhang et al.^64,66^. According to the chemical equilibria with O$^{2-}$, the concentration of electronic charge carriers, *i.e.*, electrons and holes, and thus the electronic partial conductivity $\sigma _{\textrm{e}^-}$ depends on the O$_2$ partial pressure $p_{\textrm{O}_2}$, see Fig. 3. It is the sum of the electron and hole partial conductivities, $\sigma _{\textrm{e}^\prime } + \sigma _{\textrm{h}^\cdot }$. The O$^{2-}$ partial conductivity $\sigma _{\textrm{O}^{2-}}$ does not depend on $p_{\textrm{O}_2}$ due to the high anion vacancy concentration as long as no extreme values are reached. It is identical to the oxide vacancy conductivity $\sigma _{\textrm{V}_O^{\cdot \cdot }}$. Combining the data of Park and Blumenthal with recent data for $\sigma _{\textrm{O}^{2-}}$^65^, an oxide ion transference number $t_{\textrm{O}^{2-}}$ of virtually 1 and an electronic transference number $t_{\textrm{e}^-}$ in the order of 10$^{-4}$ is obtained for an (average) O$_2$ partial pressure $p_{\textrm{O}_2}$ of 0.2 bar (air) and a temperature in the range of 800–900 $^{\circ }$C. A value in the order of 10$^{-3}$ for $t_{\textrm{e}^-}$ at 800 $^{\circ }$C (air) is reported by in the study of Zhang et al.^66^.

In the case of reduced YSZ, the number of studies is even more limited. Casselton reports an increase of the total electrical conductivity $\sigma _{\textrm{tot}}$ of 12YSZ when performing a strong electroreduction (5 A cm$^{-2}$) at 1400 $^{\circ }$C from 0.29 S cm$^{-1}$ of the pristine phase up to 4.5 S cm$^{-1}$^22^. This results in a deep blackening. The latter value may represent approximately the electronic partial conductivity $\sigma _{\textrm{e}^-}$ of the reduced phase. Bonola et al. have performed an electroreduction of 12YSZ at 600 $^{\circ }$C^26^. Heavily reduced samples exhibit an increase of $\sigma _{\textrm{tot}}$ up to 7.2 $\times$ 10$^{-2}$ S cm$^{-1}$, samples with only a light yellow colouration only up to 6.2 $\times$ 10$^{-3}$ S cm$^{-1}$ (pristine 12YSZ at 600 $^{\circ }$C^67^: $\sigma _{\textrm{O}^{2-}}$ = 4.2 $\cdot$ 10$^{-4}$ S cm$^{-1}$). Thus, the oxide ion $t_{\textrm{O}^{2-}}$ and the electronic transference number $t_{\textrm{e}^-}$ have completely changed for the reduced phase. The oxide ion transference number $t_{\textrm{O}^{2-}}$ is on the order of 10$^{-2}$ to 10$^{-4}$, presumably depending on temperature, and the electronic transference number is virtually 1. However, these estimations and calculations of $\sigma _{\textrm{e}^-}$ and $t_{\textrm{e}^-}$ are only valid since the bulk volume in these studies only consists of the reduced phase. In the case of a dendrite like morphology the real value of $\sigma _{\textrm{e}^-}$ might be even higher, as well as the value of $t_{\textrm{e}^-}$.

### Linear transport theory

The typical result of an electrochemical reduction process in a Wagner-Hebb type polarisation cell is a tongue-shaped blackened region in the center of the specimen moving from the cathode to the anode side since the surfaces of the specimen can still act as oxygen sources. Essentially, the activity of oxygen in the reduced material is controlled kinetically rather than thermodynamically. This is not only observed for YSZ, but also for barium titanate (BaTiO$_3$) and Ca-doped bismuth ferrite (BCFO, Bi$_{1-x}$Ca$_x$FeO$_{3-\delta }$)^68,69^.

**Fig. 4:**
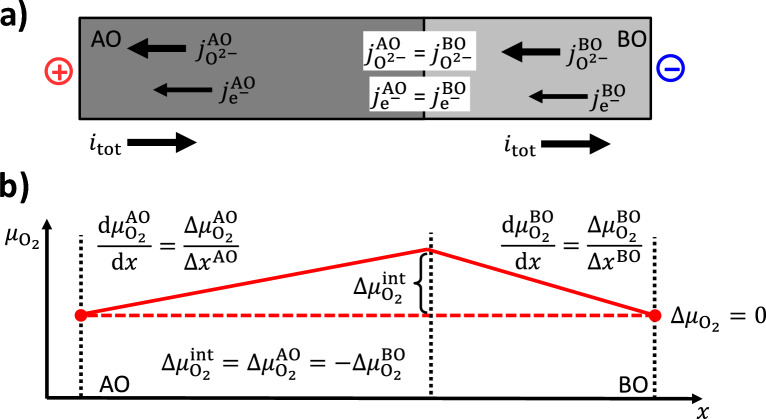
**a**) Two mixed O$^{2-}$ and e$^-$ conductors AO and BO with a common interface. An electric current $i_{tot}$ is drawn across the interface. **b**) This results to an increase of the chemical potential $\Delta \mu _{\textrm{O}_2}^{\textrm{int}}$ of O$_2$ at the interface relative to the exterior, if it is directed from AO with a higher $t_{\textrm{e}^{-}}^{\textrm{AO}}$ to BO with a lower $t_{\textrm{e}^{-}}^{\textrm{BO}}.$

**Fig. 5:**
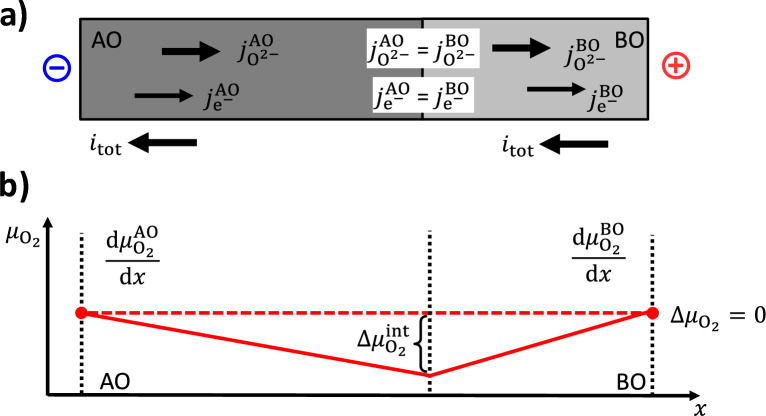
**a**) Two mixed O$^{2-}$ and e$^-$ conductors AO and BO with a common interface. An electric current $i_{tot}$ is drawn across the interface. This results to an decrease of the chemical potential $\Delta \mu _{\textrm{O}_2}^{\textrm{int}}$ of O$_2$ at the interface relative to the exterior, if it is directed from BO with a lower $t_{\textrm{e}^{-}}^{\textrm{BO}}$ to AO with a higher $t_{\textrm{e}^{-}}^{\textrm{AO}}.$

In the following, a general treatment of a static interface between two mixed oxide ions and electronic conducting phases, AO and BO, is given. There are no cation fluxes. A cell with ideally closed surfaces, *i.e.*, ion blocking electrodes and no oxygen exchange with the surrounding, is assumed for the analysis of the impact of an electric current drawn across the AO/BO interface, see Fig. 4 a) and 5 a). The two phases have different properties regarding electronic and oxide ion partial conductivity $\sigma _{\textrm{O}^{2-}}^k$ and $\sigma _{\textrm{e}^-}^k$ ($k =$ AO, BO). If there is a redox process transforming one oxide phase to the other, there will be the same cation A in both phases, but clearly distinguishable stoichiometry, structure and as well transport properties, *i.e.*, “AO” $\equiv$ AO$_{1-x}$ and “BO” $\equiv$ AO$_{1-y}$.

Linear transport theory is used to describe the oxide ion and the electronic fluxes. Vectors pointing to the right are given as positive scalar quantities and *vice versa*, as this description assumes only a one-dimensional system. The oxide ion fluxes $j_{\textrm{O}^{2-}}^{\,k}$ and the fluxes of electronic charge carriers $j_{\textrm{e}^-}^{\,k}$ in both oxide phases are given as:

1 $$\begin{aligned} j_{\textrm{O}^{2-}}^{\,k}&= -\frac{\sigma _{\textrm{O}^{2-}}^k}{(2F)^2} \frac{{\operatorname {d}\!{\tilde{\mu }_{\textrm{O}^{2-}}^k}}}{{\operatorname {d}\!{x}}} \end{aligned}$$

2 $$\begin{aligned} j_{\textrm{e}^{-}}^{\,k}&= -\frac{\sigma _{\textrm{e}^{-}}^k}{F^2} \frac{{\operatorname {d}\!{\tilde{\mu }_{\textrm{e}^{-}}^k}}}{{\operatorname {d}\!{x}}} \end{aligned}$$

Here, $\tilde{\mu }_{\textrm{O}^{2-}}^k$ and $\tilde{\mu }_{\textrm{e}^{-}}^k$ are the electrochemical potentials of oxide ions and electronic charge carriers in the oxide phases, $\sigma _{\textrm{O}^{2-}}^k$ and $\sigma _{\textrm{e}^{-}}^k$ are the oxide ion and electronic partial conductivities and *F* the Faraday constant. Assuming a static interface, *i.e.*, no running redox processes, the conservation of charge implies that the (total) electric current density as well as the oxide ion and electronic charge carrier fluxes should be equal in both phases. The difference $\Delta \mu _{\textrm{O}_2}$ of the chemical potential of oxygen at the outer boundaries is set to zero and used as reference. The electric current density $i_{\textrm{tot}}$ across the interface will induce an increase or a decrease of the oxygen potential difference $\Delta \mu _{\textrm{O}_2}^{\textrm{int}}$ of the interface to the outer boundaries depending on its direction and magnitude, see Fig. 4 b) and Fig. 5 b). A detailed treatment is given in Appendix A. It results in the following expression for $\Delta \mu _{\textrm{O}_2}^{\textrm{int}}$:

3 $$\begin{aligned} \Delta \mu _{\textrm{O}_2}^{\textrm{int}}&= -4F \frac{t_{\textrm{O}^{2-}}^{\textrm{AO}} - t_{\textrm{O}^{2-}}^{\textrm{BO}}}{\frac{t_{\textrm{O}^{2-}}^{\textrm{AO}} \sigma _{\textrm{e}^{-}}^{\textrm{AO}}}{\Delta x^{\textrm{AO}}} + \frac{t_{\textrm{O}^{2-}}^{\textrm{BO}} \sigma _{\textrm{e}^{-}}^{\textrm{BO}}}{\Delta x^{\textrm{BO}}}} i_{\textrm{tot}} \end{aligned}$$

4 $$\begin{aligned}&= 4F \frac{t_{\textrm{e}^{-}}^{\textrm{AO}} - t_{\textrm{e}^{-}}^{\textrm{BO}}}{\frac{t_{\textrm{O}^{2-}}^{\textrm{AO}} \sigma _{\textrm{e}^{-}}^{\textrm{AO}}}{\Delta x^{\textrm{AO}}} + \frac{t_{\textrm{O}^{2-}}^{\textrm{BO}} \sigma _{\textrm{e}^{-}}^{\textrm{BO}}}{\Delta x^{\textrm{BO}}}} i_{\textrm{tot}} \end{aligned}$$

The sign of the oxygen potential difference $\Delta \mu _{\textrm{O}_2}^{\textrm{int}}$ between the interface and the outer boundaries depends only on the differences $t_{\textrm{O}^{2-}}^{\textrm{AO}} - t_{\textrm{O}^{2-}}^{\textrm{BO}}$ and $t_{\textrm{e}^{-}}^{\textrm{AO}} - t_{\textrm{e}^{-}}^{\textrm{BO}}$ in the transference numbers of the charge carriers in the adjacent phases and on the direction of the electric current $i_{\textrm{tot}}$. The term in the denominator is always positive. Connecting $\Delta \mu _{\textrm{O}_2}^{\textrm{int}}$ to the oxygen partial pressure $p_{\textrm{O}_2}^{\textrm{int}}$ in the interface:

5 $$\begin{aligned} \Delta \mu _{\textrm{O}_2}^{\textrm{int}} = RT \ln \frac{a_{\textrm{O}_2}^{\textrm{int}}}{a^0_{\textrm{O}_2}} \approx RT \ln \frac{p_{\textrm{O}_2}^{\textrm{int}}}{p_{\textrm{O}_2}^0} \end{aligned}$$

the following simple rules result:

- The oxygen partial **pressure** $p_{\textrm{O}_2}^{\textrm{int}}$ in the interface will be **larger** than $p_{\textrm{O}_2}^0$, if there is an electric **current towards** the phase with the **lower electronic transference number**.
- The oxygen partial **pressure** $p_{\textrm{O}_2}^{\textrm{int}}$ in the interface will be **smaller** than $p_{\textrm{O}_2}^0$, if there is an electric **current towards** the phase with the **higher electronic transference number**.

The oxygen activity and partial pressure at the outer boundaries are denoted as $a^0_{\textrm{O}_2}$ and $p_{\textrm{O}_2}^0$.

## Experimental setup

**Fig. 6:**
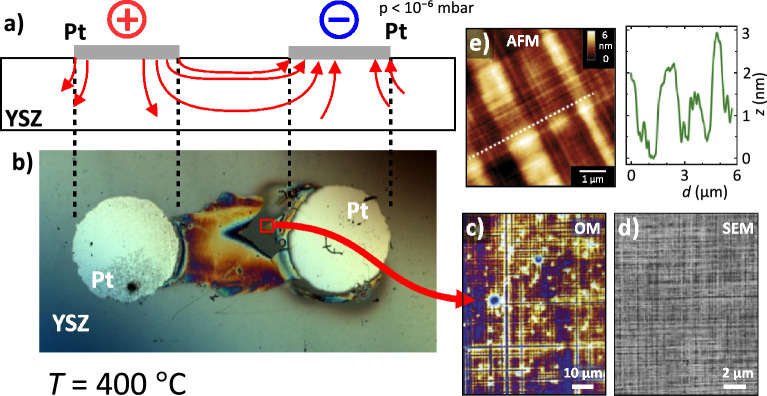
Surface investigation of the electroreduced YSZ sample. **a**) Cross-section illustration of the experimental setup with the Pt electrodes shown in grey and the flow of oxygen ions following the electric field in the sample as red arrows. **b**) Optical micrograph of the sample after electroreduction in top-view. **c**) Detailed investigation of the heavily reduced zone phase contrast optical microscopy (OM), **d**) scanning electron microscopy (SEM), and **e**) atomic force microscopy (AFM) with topography profile measured along the dashed line. Adapted from Rodenbücher et al.^38^.

The electro-colouration experiment is performed on single crystalline YSZ as described in detail in a previous publication^38^. A commercial ZrO$_2$ single crystal doped with 9.5 mol% Y$_2$O$_3$ grown using the skull crucible process are used (Crystec, Berlin, Germany). The sample is cut to a size of 10 $\times$ 3 $\times$ 0.5 mm${^3}$ by a diamond saw. The surface is epipolished and parallel to (100) YSZ. Two circular platinum electrodes with a diameter of approximately 2 mm, a thickness of approximately 100 nm and a distance of 2.2 mm are deposited by cathode sputtering on the surface, see also Fig. 6a. Electroreduction process is performed in a vacuum chamber at a base pressure of approximately 10$^{-6}$ mbar maintained by a turbomolecular pump (Leybold, Cologne, Germany) to prevent reoxidation. The sample’s temperature is hold at 400 $^{\circ }$C by an electric heater. The experiment is conducted at a voltage of 1 kV applied to the electrodes and the resulting current is monitored (BOP 1000 M, Kepco, Flushing, USA and SourceMeter 6430, Keithley, Solon, USA). A current compliance of 10 mA is established by a series resistor of 100 k$\Omega$ to prevent premature disruption of the crystal. The duration of the experiment is approximately 16 h. In general, as soon as the decomposition voltage of 2.47 V is exceeded, the oxygen activity at the cathode would be so low that electroreduction starts and new phases can form. The value is obtained when considering the thermodynamic equilibrium between ZrO$_2$ and metallic Zr-metal at the given temperature. However, because of the low temperature, this transformation reaction will take place on a long timescale, as the mobility/partial conductivity of oxide ions is only low. Hence, we used a high voltage to accelerate the electroreduction by increasing the driving force for ionic transport and to be able to observe the degradation effects in a reasonable experimental time.

### Optical microscopy, SEM and AFM

The surface of the reduced samples are investigated by phase contrast light microscopy (PCM, Axio Imager, Zeiss, Germany) and scanning electron microscopy (SEM, Quanta 3D FEG, FEI, Hillsboro, USA), using an acceleration voltage of 5 kV and backscattered electron (BSE) imaging mode. Selected regions of the surface are characterised by atomic force microscopy (AFM, Cypher S, Asylum Research, USA).

### STEM, TEM/SAED, EDX and EELS

To get more insights, cross-sections in selected regions of the surface are prepared for TEM investigations by using a gallium focused ion beam gun (FIB). The microstructural studies using transmission (TEM) and scanning transmission electron microscopy techniques (STEM), at an acceleration voltage of 300 kV, are carried out using an FEI Titan G2 instrument (Thermofisher, USA), equipped with an X-FEG field emitter, a ChemiSTEM X-ray spectrometer (EDX) and a Gatan GIF Quantum 963 electron energy loss spectrometer (EELS).

Using EDX, information on the local chemical composition regarding zirconium, yttrium, and oxygen can be obtained, using the Zr-K$_{\mathrm {\alpha }}$ (15.69 and 15.77 keV), Y-K$_{\mathrm {\alpha }}$ (14.88 and 14.96 keV) and O-K$_{\mathrm {\alpha }}$ (0.525 keV) lines. The crystallographic structure and the mutual orientation of the reduced and unreduced phase is investigated by selected area diffraction (SAED) and by Fourier analysis of high-resolution images. The fine structures of the electron energy loss near edge structure (ELNES) reflecting the chemical state of the constituent atoms are studied using EELS technique for Zr-L$_{2,3}$,Y-L$_{2,3}$, and O-K edges.

## Results

### Optical microscopy, electric resistance, SEM, and AFM

Starting from the cathode, a blackened zone is spreading to the anode side. For the chosen geometry and voltage, the current increases and reaches the selected compliance of 10 mA after 170 s. The experiment is continued for 16 h at a constant current of 10 mA. The current compliance, accompanied with a very low sample resistance, marks the end of the reduction process, as a “quasi” short circuit appears. During the reduction process the measured current varies by 5–6 orders of magnitude, resulting in a decrease of the cell resistance from 10$^{11}$ $\Omega$ in the beginning to below 1.8 k$\Omega$. For details, see Rodenbücher et al.^38^. Apparently, the diffuse reaction front is reaching the anode just before a strong drop in electrical resistance is observed. This indicates that there is at least more than one stage of reduction.

Fig. 6 shows an illustration of the flow of oxygen ions during the electro-reduction and a detailed analysis of the sample’s surface after completion of the experiment. Close to the cathode, a heavily reduced, deeply blackened area can be observed by light microscopy, see Fig. 6b). A peculiar “checkerboard” structure can be identified in this area, see Fig. 6c). Limited by the resolution of this method, perpendicular “rips” with the order of 2 µm can be observed. The presence of the “checkerboard” structure in the heavily reduced area can be verified by SEM and AFM. The average distance of the “ribs” can be determined more precisely to 1–2 µm, see Fig. 6d) and e). They are well aligned to the main axes $\langle 100\rangle$ and $\langle 010\rangle$ of the cubic YSZ (edges of the YSZ substrates). Using AFM, it is also possible to determine the height of the “ribs” as additional information. An average value of 1–2 nm can be measured, see the line profile in Fig. 6e).

### STEM, TEM/SAED, EDX and EELS

**Fig. 7:**
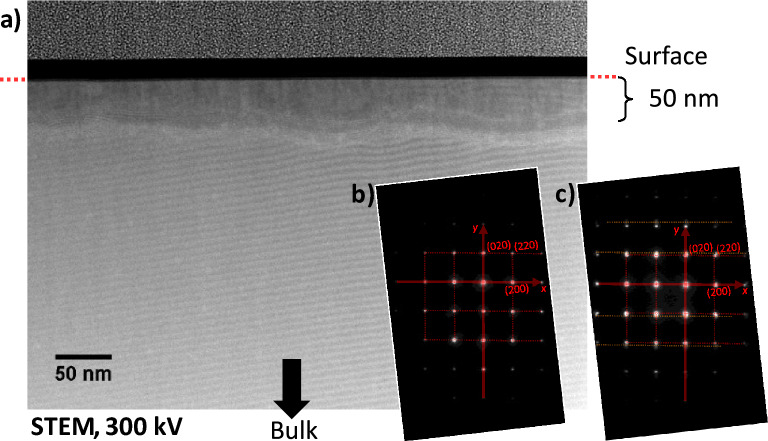
STEM investigation (300 kV) of a sample, cross sectioned in the heavily reduced area at the cathode side. **a**) Micrograph of a region close to the surface using the high-angle annular dark field (STEM/HAADF) mode. SAED of the bulk region **b**) and of a region **c**) including the “belt-shaped” feature 50 nm below the surface.

Figure 7 shows an image of the cross section of the reduced area near the cathode obtained by Scanning Transmission Electron Microscopy in high-angle annular dark field (STEM/HAADF) mode. A “belt shaped” feature can be observed about 50 nm below the sample surface, see Fig. 7 a), which clearly is distinguishable from the surrounding bulk. The “belt shaped” feature has a thickness of 20 to 30 nm (perpendicular to the surface). When recording an SAED from the bulk region below the “belt” or from the region directly situated between the surface and the “belt”, a diffraction pattern as expected for a cubic material is obtained, see Fig. 7 b). The beam is directed along a fourfold main axis $\langle 001\rangle$. When recording a SAED of a region including the “belt-shaped” feature, a set of additional reflections appears, elongated along $\langle 010\rangle$, *i.e.*, perpendicular to the sample surface plane, see Fig. 7 c).

**Fig. 8:**
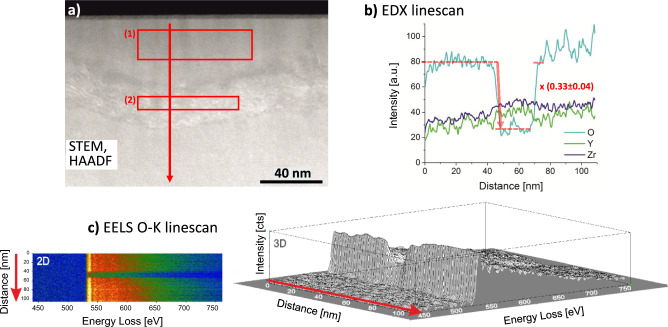
EDX/STEM investigation of a sample, cross sectioned in the heavily reduced area at the cathode side. **a**) Micrograph of a region close to the surface with the “belt-shaped” feature. The red regions (1) and (2) mark the EDX analysis regions (cf. Table 2). **b**) EDX linescan for the elements Zr, Y and O across the “belt-shaped” feature. **c**) background removed EELS oxygen K linescan (in 2D and 3D representations) across the “belt-shaped” feature taken along the the red marked arrow in (**a**).

Fig. 8 shows a combined EDX and EELS analysis of the “belt shaped” feature in the cross-section. A strong oxygen deficit in the heavily reduced area close to the cathode compared to the pristine composition of 9.5YSZ can be observed by EDX, see Fig. 8 a). A region (1) above the “belt-shaped” feature and (2) within the feature is analysed, see red boxes in Fig. 8 a). The Cliff-Lorimer method (thin-film approximation) is used for the evaluation of the data, see Table 2. Note that, due to the limitations of the applied method, the proposed stoichiometries are approximate and should be interpreted as semi-quantitative estimates. Despite these limitations, STEM/EDX remains a valuable tool for identifying relative compositional changes at the nanoscale, particularly when combined with high-resolution structural imaging^70^. Hence, we employ STEM/EDX here to provide relative, comparative information on oxygen depletion (trends). The largest deficit is found in the region (2) within the “belt-shaped” feature. Remarkably, when normalising the total metal content to unity, for both analysed regions, the measured atomic ratio between Zr and Y does not fit to the nominal composition of 9.5YSZ given by the supplier, *i.e.*, (Zr$_{0.83}$Y$_{0.17}$)O$_{1.91}$. A composition of (Zr$_{0.79}$Y$_{0.21}$)O$_{0.50}$ can be found in region (1) above the “belt-shaped” feature and of (Zr$_{0.81}$Y$_{0.19}$)O$_{0.12}$ in region (2) within the “belt-shaped” feature. This would rather correspond to an actual Y$_2$O$_3$ content of 11 mol% of the pristine material, *i.e.*, (Zr$_{0.80}$Y$_{0.20}$)O$_{1.90}$. Compared to the oxygen atom content of 65.5 at% of the pristine 11YSZ, the oxygen content in the region (1) above the “belt-shaped” feature corresponds to a factor of 1/2 and in the region (2), located within the feature, even to a factor of less than 1/6 of the original value. This fits to the decrease of the O-K$_{\mathrm {\alpha }}$ signal in the EDX linescan to a value of 1/3, see Fig. 8 b). One also needs to take into account that the TEM analysis has to be regarded as a *post mortem* method. Hence, a slight reoxidation of the sample due to contact with ambient air cannot be excluded and the oxygen content directly after the electroreduction process could even be lower.

In addition to the EDX results, the EELS studies reveal a strong oxygen deficit, depicted in Fig. 8 c), which corresponds to the thickness of the “belt-shaped” feature. A slight difference in the scattering properties can also be noticed for distinguished regions along the O-K linescan. At the Zr-L$_{2,3}$ edge, a chemical shift of the Zr-L$_3$ onset is visible and corresponds to approximately +1.0 eV for the “belt region” as compared to the ZrO$_2$ bulk signal. This edge shift is accompanied by a simultaneous decrease of the Zr-L$_{2,3}$ branching ratio BR=I(L$_3$)/(I(L$_3$)+I(L$_2$)) suggesting an intermediate oxidation state for the “belt region” between metallic Zr and bulk ZrO$_2$. A similar behaviour can be noticed for Y-L$_{2,3}$ edge suggesting formation of a suboxide phase upon heavily reduction within the “belt region”. Although the applied approach does not allow a definitive assignment of formal oxidation states we can conclude that the “belt-shaped” feature has Zr and Y in a suboxide state with approximated oxidation states between 0 and + II. However, for a definitive proof for the Zr and Y oxidation states, further work (with standards and high spatial and energy resolution experiments) would be required.

**Table 2:** Results of the EDX investigation for the elements Zr, Y and O of the two regions above (1) and within (2) the “belt-shaped” feature depicted in Fig. 8a. The Cliff-Lorimer method (thin-film approximation) is used for the evaluation of the data.

Element	Series	wt.%	Error wt.%	at.%
Region (1), **above** the “belt-shaped” feature:				
Zr	K$_\alpha$	73.4	±2.3	52.9
Y	K$_\alpha$	18.5	±0.7	13.7
O	K$_\alpha$	8.1	±0.3	33.3
Sum:		100		100
$\rightarrow$(Zr$_{0.79}$Y$_{0.21}$)O$_{0.50}$				
Region (2), **within** the “belt-shaped” feature:				
Zr	K$_\alpha$	79.3	±2.7	72.2
Y	K$_\alpha$	18.7	±0.8	17.4
O	K$_\alpha$	2.0	±0.1	10.4
Sum:		100		100
$\rightarrow$(Zr$_{0.81}$Y$_{0.19}$)O$_{0.12}$				

**Fig. 9:**
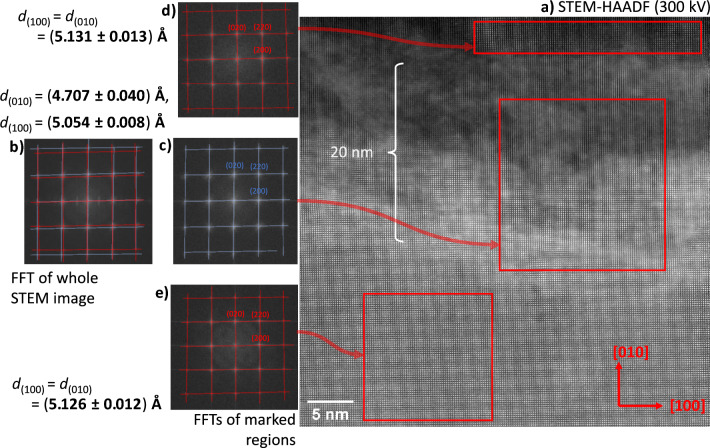
STEM investigation (300 kV) of a sample, cross sectioned in the heavily reduced zone at the cathode side. **a**) Micrograph of a region close to the surface using HAADF mode at atomic resolution. **b**)–**d**) FFTs of the (red) marked regions and of the whole micrograph.

A STEM/HAADF micrograph of the “belt-shaped” feature, its interfaces with the surrounding bulk and the bulk above and below is depicted in Fig. 9 a). A fast Fourier transform (FFT) algorithm is applied to selected regions using the ImageJ software^71^. An FFT of the whole micrograph, see Fig. 9 b), yields the same result as obtained by SAED of a region including the “belt-shaped” feature, *i.e.*, two sets of reflections, the second set elongated along the $\langle 001\rangle$ direction. Within the “belt-shaped” feature, only the set of reflections is obtained, elongated along the $\langle 010\rangle$ direction, see Fig. 9 c). The axis in beam direction has only a two-fold symmetry. When evaluating FFTs from regions outside the “belt-shaped” feature, see Fig. 9 d) and e), sets of reflections are obtained according to the structure of a cubic material. The axis in beam direction has a four-fold symmetry.

The calibration of the instrument, *i.e.*, the nm bar, was performed by analysing a micrograph of a gold nanoparticle and of the phase above the “belt-shaped” feature by using the same instrumental parameters, see “Supplemental Material”, 1). Thus, the absolute values of the $\vec {g}$ vectors of the lattice planes can be measured from the FFTs, see “Supplemental Material”, 2) to 4).

## Discussion

### Electrical properties

From the strong drop in the sample’s electric resistance by 7–8 orders of magnitude during electroreduction, one can conclude that the strongly reduced phase has semiconducting or even metallic properties. Considering the sample geometry between the electrodes, an electronic conductivity $\sigma _{\textrm{e}^-}$ on the order of 10$^{-9}$ S cm$^{-1}$ can be estimated for the pristine 11YSZ and a value on the order of 10$^{-2}$ S cm$^{-1}$ for the reduced material. A value on the order of 10$^{-9}$ S cm$^{-1}$ for the pristine 11YSZ is in principle reasonable when keeping in mind the different doping degree and the underestimated cross-sectional area, see Fig. 3^64^. Due to the preparation method (skull melting) of YSZ single crystals at high temperature and ambient air and the subsequent cooling down, the final stoichiometry will be “frozen-in” at a temperature the oxygen diffusion becomes too slow. This may take place below approximately 500 $^{\circ }$C. The experimental temperature of 400 $^{\circ }$C is far too low for a fast equilibration of the sample to the ambient oxygen partial pressure in the vacuum chamber. At a $p_{O2}$ of 0.2 bar and 400 $^{\circ }$C a hole partial conductivity of $10^{-9}$ to $10^{-10}$ S cm$^{-1}$can be extrapolated. As the new phase can be observed approximately 60 nm below the sample surface, forming a “belt-shaped” morphology, the conductivity within a cross-section of the reduced sample is most probably very inhomogeneous and rather localised to certain regions. A dendrite-shaped morphology of the reduced region is known from preceding studies^31,38^. Thus, the estimated conductivity of 10$^{-2}$ S cm$^{-1}$is only a lower limit. Bonola et al. have measured comparable values^26^.

Considering the thermodynamic data of Zr metal, O$_2$ and $\alpha$ZrO$_2$ at $T =$ 500 $^{\circ }$C, a free formation enthalpy $\Delta _{\textrm{f}} G^0(\alpha \text {ZrO}_2)$ of −953 kJ mol$^{-1}$is obtained^72^. Thus, the presence of metallic Zr as reduced phase would result in an equilibrium O$_2$ activity of $\lg a_{\textrm{O}_2}$ = −64. In an ideal Wagner-Hebb polarisation cell with an O$_2$ permeable anode in equilibrium with air^1^, this corresponds to a cell potential of *U*= 2.47 V^73,74^. This is the lower limit to start an electrochemical reduction process. A higher voltage will speed it up, because of the low ionic conductivity at 500 $^{\circ }$C.

Considering the own results and the values reported in the literature on the electronic conductivity of the reduced phase, a reverse of the ionic and electronic transference numbers after reduction can be assumed. The difference in transference numbers $t_{\textrm{e}^{-}}^{\mathrm {\,red.YSZ}} - t_{\textrm{e}^{-}}^{\textrm{YSZ}}$ is about 1. Thus, a current directed from the unreduced to the reduced phase will decrease the oxygen potential $\Delta \mu _{\textrm{O}_2}^{\textrm{int}}$ at the interface, see Eq. (4). Due to its high electronic transference number, the reduced phase will act as a moving quasi-metallic cathode.

There is no sharp transition between the largely ionic conducting unreduced phase and the largely electronic conducting reduced phase. Taking into account the oxygen potential dependence of the conductivities in Fig. 3, it is rather broadened over a distinct space.

### Structure and composition of the new phases

The results for the composition and the lattice spacings in the heavily reduced area close to the surface at the cathode side deviate strongly from the unreduced pristine YSZ, see Table 2, Fig. 8 and 9.

#### Above and below the “belt-shaped” feature

The observed regular ripple structure in the STEM/HAADF micrograph is mainly caused by the electron density variations of lattice planes occupied by Zr and Y cations, since the highest electron density is accompanied with these atoms, see, Fig. 9 a). It can be assigned to (200) and (020) lattice planes. The orthogonal patterns with four-fold symmetry found in the FFTs and the SAED of the regions below and above the “belt-shaped” feature are in accordance with an Fm$\bar{3}$m material as bulk YSZ with an fcc arrangement of the cations and a beam direction along the main axis [001], see Fig. 9 d), e) and Fig. 7 b). The strongest signals in the FFT pattern can be identified as (200), (020), ($\bar{2}$00),(0$\bar{2}$0), (220), (2$\bar{2}$0), ($\bar{2}$20) and ($\bar{2}\bar{2}$0).

The spacings of the (100) and (010) planes evaluated from the FFTs yield a value of (5.128 ± 0.009) Å for the region below and above the “belt-shaped” feature (as an average). There is no significant difference between the lattice constants *a* and *b*. The structural characterisation is still incomplete, as the change of the spacing of the (001) planes, perpendicular to the beam direction, cannot be determined. The new phase has either a cubic structure:

$$a = b = c = 5.128$$ Å, $$\alpha = \beta = \gamma =$$ 90$$^{\circ }$$

or a tetragonal structure, $a = b \ne c$. A cubic structure corresponds to a contraction of the lattice constant *a* by 0.37% and of the unit cell volume by 1.1%, compared to pristine bulk 11YSZ, $a =$ (5.147 ± 0.003) Å, see “Supplemental material”, 4).

The STEM/EDX analysis reveals a strong oxygen deficiency. According to the results in Table 2 and the linescan in Fig. 8 b), the material above and below the “belt-shaped” feature has a stoichiometry of (Zr$_{0.79}$ Y$_{0.21}$)O$_{0.50}$, *i.e.*, (Zr$_{0.79}$ Y$_{0.21}$)$_2$O (within the limits of the applied method). The stoichiometries for various possible reduction stages of 11YSZ are exemplarily calculated and compiled in Table 3. The first bold values table entry fits best. The oxygen deficit found is only possible, if mainly Zr$^\textrm{I}$ and Y$^\textrm{I}$ are present.

**Table 3:** Calculated compositions of reduced 11YSZ, assuming a possible presence of Zr$^\textrm{IV}$, Zr$^\textrm{II}$, Zr$^\textrm{I}$, Zr$^\textrm{0}$, Y$^\textrm{III}$, Y$^\textrm{II}$, Y$^\textrm{I}$ and Y$^\textrm{0}$, but only a single oxidation stage per element.

Molar fractions of oxides					Metal content normalised to 1			at%		
					Zr	Y	O	Zr	Y	O
0.89	Zr$^\textrm{IV}$O$_2$	+	0.11	Y$^\textrm{III}_2$O$^{}_3$	0.802	0.198	1.901	27.6	6.8	65.5
0.89	Zr$^\textrm{II}$O	+	0.11	Y$^\textrm{III}_2$O$^{}_3$	0.802	0.198	1.099	38.2	9.4	52.4
0.89	Zr$^\textrm{II}$O	+	0.11	$\cdot$ 2 Y$^\textrm{II}_{}$O	0.802	0.198	1.000	40.1	9.9	50.0
0.89	Zr$^\textrm{II}$O	+	0.11	Y$^\textrm{I}_2$O	0.802	0.198	0.901	42.2	10.4	47.4
0.89	Zr$^\textrm{II}$O	+	0.11	$\cdot$ 2 Y$^\textrm{0}_{}$	0.802	0.198	0.802	44.5	11.0	44.5
0.89	$\cdot$ 1/2 Zr$^\textrm{I}_2$O	+	0.11	Y$^\textrm{III}_2$O$^{}_3$	0.802	0.198	0.698	47.2	11.7	41.1
0.89	$\cdot$ 1/2 Zr$^\textrm{I}_2$O	+	0.11	$\cdot$ 2 Y$^\textrm{II}_{}$O	0.802	0.198	0.599	50.1	12.4	37.5
**0.89**	$\boldsymbol{\cdot }$ **1/2 Zr**$^{{\textbf {I}}}_{\boldsymbol{2}}$**O**	**+**	**0.11**	${\textbf {Y}}^{{{\textbf {I}}}}_{{\boldsymbol{2}}}$**O**	**0.802**	**0.198**	**0.500**	**53.5**	**13.2**	**33.3**
0.89	$\cdot$ 1/2 Zr$^\textrm{I}_2$O	+	0.11	$\cdot$ 2 Y$^\textrm{0}_{}$	0.802	0.198	0.401	57.2	14.1	28.6
0.89	Zr$^\textrm{0}$	+	0.11	Y$^\textrm{III}_2$O$^{}_3$	0.802	0.198	0.297	61.8	15.3	22.9
0.89	Zr$^\textrm{0}$	+	0.11	$\cdot$ 2 Y$^\textrm{II}_{}$O	0.802	0.198	0.198	66.9	16.5	16.5
**0.89**	${\textbf {Zr}}^{{{\textbf {0}}}}$	**+**	**0.11**	${\textbf {Y}}^{{\textbf {I}}}_{{\boldsymbol{2}}}$**O**	**0.802**	**0.198**	**0.099**	**73.0**	**18.0**	**9.0**
0.89	Zr$^\textrm{0}$	+	0.11	$\cdot$ 2 Y$^\textrm{0}_{}$	0.802	0.198	0.000	80.2	19.8	0.0

The composition found corresponds to the metastable Zr$_2$O phase with a cubic Cuprite structure (Pn$\bar{3}$m), reported by Khitrova and Klechkovskaya and Henning et al.^10,52^. This would fit to an unchanged fcc arrangement of the cations. The reported lattice constant of *a* = 5.088 Å is comparable to the measured value, actually it is only 0.78% smaller, considering a general increase of the lattice parameters due to the Y$_2$O$_3$-doping, see “Supplemental material”, 5). However, in the case of a cuprite structure, additional (110), ($\bar{1}$10), (1$\bar{1}$0) and ($\bar{1}\bar{1}$0) reflections should be visible in the SAED (and in the FFTs) due to the non-random distribution of the oxygen ions, see Fig. 7 b) and Fig. 9 e) and d). The absence may indicate a fluorite structure with the cations on 4a sites and a random distribution of the oxygen ions on the 8c sites (1/4 occupation).

#### Within the “belt-shaped” feature

According to the STEM/EDX analysis, the oxygen content of the material within the “belt-shaped” feature is even lower compared to the phase above and below, see Table 2. A stoichiometry of (Zr$_{0.81}$ Y$_{0.19}$)O$_{0.12}$ is found, *i.e.*, (Zr$_{0.79}$ Y$_{0.21}$)$_{8.6}$O. The Zr/Y cation ratio is approximately the same as in the phase above and below. At the experimental temperature, it can be safely assumed that there is no cation mobility^75^. The oxygen deficit indicates an average oxidation state below +I. A presence of Zr$^\textrm{0}$ and Y$^\textrm{I}$ is roughly in accordance with the measured composition, see second bold values in Table 3. Such a high metal content would correspond to Zr-O intercalation compounds such as $\alpha _1^{\prime \prime }$Zr or $\alpha _2^{\prime \prime }$Zr based on the hcp structure of metallic Zr. It should be kept in mind that a detailed structural analysis of the new phase in the “belt-shaped” feature by XRD is not feasible (even using synchrotron beamlines) as the X-ray beam is of the size of the whole sample volume. Only TEM/electron diffraction allows for the necessary spatial resolution.

The SAED and FFTs, taken from regions within the “belt-shaped” feature, exhibit a stretched orthogonal pattern with only a two-fold symmetry, see Fig. 7 c) and Fig. 9 c). Compared to the phase below and above the “belt-shaped” feature, there is a contraction along the [010] direction, *i.e.*, perpendicular to the surface.

An hcp arrangement of Zr (and Y), as found in Zr-O intercalation compounds, may also lead to such orthogonally stretched SAED/FFT patterns, if the beam is parallel to [120]. However, analysing the absolute values of the $\vec {g}$ vectors in the FFTs for such orthogonal patterns, the ratio of the lattice spacings does not fit to a hcp structure, *i.e.*, $d_{(0002)}/d_{(11\bar{2}0)} \approx$ 1.6. Moreover, there is no sharp boundary to the phase above and below. The observed regular ripple structure along the [100] direction in the STEM/HAADF micrograph due to the electron density variations is aligned between both phases. Some misfit dislocations exist, see below. Thus, an hcp arrangement of the Zr (and Y) atoms is quite unprobable. Presumably, a distorted fcc arrangement is present.

These considerations would imply an orthorhombic or tetragonal structure and three 90$^{\circ }$ angles in the unit cell. The analysis of the $\vec {g}$ vectors in the FFTs results in a value of (5.054 ± 0.008) Å for the (100) spacings and of (4.707 ± 0.040) Å for the (010) spacings. This is a distortion of 6.9%. Such a distortion cannot be explained only by a strain state. Hence, a new face-centred orthorhombic distorted structure may be present:

$$a \approx$$ 5.054 Å, $$b \approx$$ 4.707 Å, $$c =~?$$ Å,

$$\alpha = \beta = \gamma =$$ 90$$^{\circ }$$

or one of two possible body-centred tetragonal distorted structures (original unit cell to be halved):

$$a^\prime = b^\prime \approx \frac{\sqrt{2}}{2}$$ 5.054 Å, $$c^\prime \approx$$ 4.707 Å,

$$\alpha ^\prime = \beta ^\prime = \gamma ^\prime =$$ 90$$^{\circ }$$

$$a^{\prime \prime } = b^{\prime \prime } \approx \frac{\sqrt{2}}{2}$$ 4.707 Å, $$c^{\prime \prime } \approx$$ 5.054 Å,

$$\alpha ^{\prime \prime } = \beta ^{\prime \prime } = \gamma ^{\prime \prime } =$$ 90$$^{\circ }$$

If an orthorhombic structure is present, one lattice constant (*c*) cannot be determined, as the corresponding lattice axis is parallel to the beam direction. Assuming a tetragonal distortion, this would result in a volume contraction of the phase in the “belt-shaped” region of 11 to 17% compared to the phase above and below and 12 to 18% compared to pristine 11YSZ. A structure with a distorted fcc arrangement of Zr (and Y) does not correspond to any known Zr-O intercalation compound based on an hcp structure of metallic Zr. It is not clear whether the oxygen ions are randomly distributed or ordered, due to their low concentration and the very different scattering factors of Zr, Y and O.

### Mismatch with bulk phase, strain relaxation

Investigations using TEM/SAED and STEM/FFT on the “belt-shaped” phase and the phase above and below in the region close to the cathode side indicate a strong structural distortion of the new phases to each other. There is a contraction of the lattice spacings of 1.4% in [100] direction and of 8.1% in the [010] direction, *i.e.*, perpendicular and parallel to the sample surface plane. The lattice mismatch in the [100] direction between the “belt-shaped” phase and the phase above and below results in mismatch dislocations in the interface due to strain release.

By performing a geometric phase analysis (GPA), it is possible to extract the distortion field of the atomic lattice and finally the strain field (components of the tensor: $\varepsilon _{xx}$, $\varepsilon _{yy}$ and $\varepsilon _{xy}$). The lattice compression/elongation in a particular direction is described in a relative way^76,77^.

**Fig. 10:**
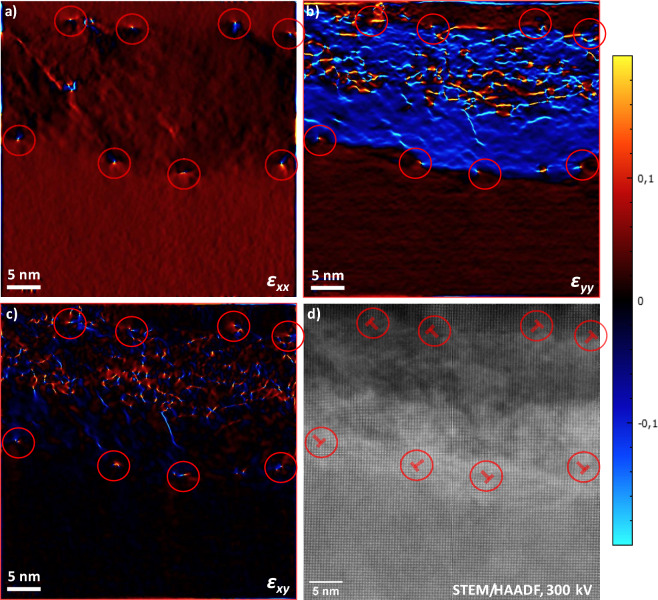
Geometric Phase Analysis **a**) – **c**) of the STEM/HAADF micrograph a), also depicted in Fig. 9. The (200) and (020) reflections of the FFT are considered. The components $\varepsilon _{xx}$, $\varepsilon _{yy}$ and $\varepsilon _{xy}$ of the strain tensor are depicted. Mismatch dislocations in the interface between “belt-shape” phase and the phase above and below are marked with circles and $\bot$.

In Fig. 10 a) – c) a GPA of the STEM/HAADF micrograph, depicting a cross section in the heavily reduced zone at the cathode side, see also Fig. 9 a). Considering the calculated strain tensor components $\varepsilon _{xx}$ and $\varepsilon _{yy}$, it can be readily seen that a compressive distortion is present in the “belt-shaped” phase to the phase above and below, mainly in the [010] direction.

At the interfaces between the “belt-shape” phase and the phase above and below, local dipole-type strain fields can be identified for all depicted strain tensor components. For the $\varepsilon _{xx}$ component, the compressive pole is always directed to the “belt-shaped” phase and the dilative pole the phase above and below. In the case of the $\varepsilon _{yy}$ component, the strain field dipoles are roughly orientated parallel to the surface plane, respectively to the interface plane.

**Fig. 11:**
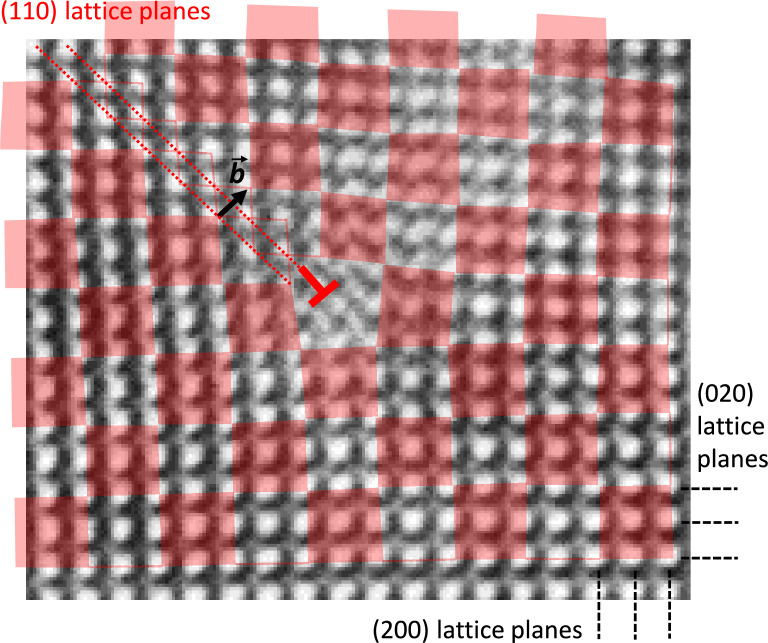
Detail of Fig. 10 d) at the location of the left-most localised dipole strain field. The Burgers vector $\vec {b}$ of a fcc lattice-type edge dislocation is marked with a black arrow, the extra (110) planes with hashed red lines. The unit cells are indicated with transparent red squares.

A detail of Fig. 10 d) with a local dipole strain field is depicted in Fig. 11. It coincides with the ends of two extra (110) lattice planes. Thus, an arrangement of misfit dislocations can be identified in the interfaces. An edge dislocation in an (ideal) fcc lattice is aligned to the $\langle 110\rangle$ directions as the {111} planes are the slip planes. The burgers vectors in the “bet-shaped” phase are orientated in a 45$^{\circ }$ angle to the interface plane, *i.e.*, $\vec {b} = a/2\,[110]$.

These edge dislocations may be present due to a strain release in [100] direction parallel to the sample surface. Considering the (100) lattice plane spacings of the “belt-shaped” phase and the phase above and below of (5.054 ± 0.008) and (5.128 ± 0.009) Å, a misfit $f = \Delta a/a$ of (1.44 ± 0.33)% can be calculated. The mismatch strain is compensated for by its localisation in dislocations in the interface. The necessary average dislocation spacing *D* in [100] direction for full strain release can be calculated according Sutton and Baluffi^78^:

6 $$\begin{aligned} D = \frac{b_{[100]}}{f} \end{aligned}$$

Here, $b_{[100]}$ is the projection of the burgers vector $\vec {b}$ on the [100] direction. Considering the measured lattice constants, a value of *a*/2 = (2.527 ± 0.004) Å can be calculated. This results in a distance *D* of about (17.6 ± 8.1) nm, which fits very roughly to the observed average spacing in the investigated image region, considering the high error margins as small differences of values with considerable errors are calculated. The “chequerboard” structure may be the result of a gliding of edge dislocations from the surface to the interface of the “belt-shaped” phase as a consequence of strain relaxation.

In the upper half of the “belt-shape” phase, a large number of strip-shaped strain fields can be identified only for the $\varepsilon _{xx}$ and $\varepsilon _{xy}$ component. There is no $\varepsilon _{xx}$ component. This indicates the presence of many stacking faults.

According to these results, the deterioration of the mechanical properties of electrochemical reduced YSZ, often reported in the literature, may be induced by the generation of internal strain originated from the strong volume contraction of the new phases relative to the unreduced YSZ, respectively to each other.

## Conclusions

Yttria stabilised zirconium dioxide (Zr,Y)O$_2$ can be electrochemically reduced in a Wagner-Hebb-type polarisation cell under vacuum conditions. A reduced substoichiometric phase (Zr,Y)O$_{2-x}$ is growing from the cathode to the bulk. The reduced phase has a highly increased electronic partial conductivity and transference number. The change in the electronic transference numbers of the two adjacent phases, *i.e.*, the reduced and unreduced oxide, will unavoidably lead to persisting oxygen potential gradients and to a proceeding reduction reaction at the mutual interface, when drawing a current. As a result, a reduction front will shift through the bulk.

At the cathode side and close to the sample surface, new possibly metastable phases with strong oxygen deficit are found. Starting from a nominal composition Zr$_{0.80}$Y$_{0.20}$O$_{1.93}$ (11YSZ), reduced phases with a composition of (Zr$_{0.79}$Y$_{0.21}$)O$_{0.50}$ and (Zr$_{0.81}$Y$_{0.19}$)O$_{0.12}$, *i.e.*, (Zr,Y)$_2$O and (Zr,Y)$_{8.6}$O are formed. This indicates the presence of mainly Zr$^\text {I}$ and Y$^\text {I}$, respectively of Zr$^\text {0}$ and Y$^\text {I}$. EELS studies of Zr-L$_{2,3}$ and Y-L$_{2,3}$ edges advocate for the suboxide phase formation upon heavily reduction within the “belt region” with a Zr oxidation state between 0 and +II. The new phases have a significantly smaller molar volume compared to unreduced 11YSZ. There is a strong contractile distortion perpendicular to the interface.

It is not possible to identify these phases according to the present structural and compositional data in the literature. Most probably, (Zr,Y)$_2$O has a cubic face centred arrangement of Zr and Y and randomly distributed O on the 8c positions. In the case of (Zr,Y)$_{8.6}$O, a tetragonal or orthorhombic distorted face centred arrangement of Zr and Y and randomly disordered O on the originally 8c sites can be assumed. According to theoretical calculations by Zhang et al. on Zr$_6$O, Zr$_3$O and Zr$_ 2$O, it can be assumed that the metal rich phase (Zr,Y)$_2$O and (Zr,Y)$_{8.6}$O also exhibit metallic conductivity^60^. The presence of these phases may be responsible for the very high conductivity of strongly reduced YSZ.

A strong volume decrease results in strain not only between the newly found phases but also between the heavily reduced new phases and the more slightly reduced surrounding YSZ. The release of the strain leads to a regular arrangement of mismatch dislocations between the reduced phases and may also be the cause for the prominent “chequerboard” structure on the surface above, due to a gliding of edge dislocations from the surface. This may also be the reason for the often observed deterioration of the mechanical properties of electrochemical reduced YSZ. In the literature it is indicated, that there are more than one reduction stages. Possibly, the newly identified phases represent the final stages of electrochemical reduction, starting from coloured YSZ by formation of only F-centres.

## Supplementary Information

Supplementary Information.

## Data Availability

The datasets generated during and/or analysed during the current study are available from the corresponding author on reasonable request.

## A Treatment of the polarisation process by linear transport theory

Based on the assumptions in section Linear transport theory, a general treatment of an interface between two mixed oxide ions and electronic conducting phases AO and BO is given by using linear transport theory. For the sake of simplicity, the following description assumes only a one-dimensional system, *i.e.*, vectors pointing to the right are given as positive scalar quantities and *vice versa*, see Fig. 4 a). The oxide ion flux $j_{\textrm{O}^{2-}}^{\,k}$ and the electronic charge carrier flux $j_{\textrm{e}^-}^{\,k}$ in the oxide phases are given in Eq. 1 and 2 ($k =$AO, BO). Assuming no redox reactions, the conservation of charge and implies the following boundary condition for the total electric current density $i_\textrm{tot}$, the oxide ion $j_{\textrm{O}^{2-}}^{\,k}$ and electronic partial fluxes $j_{\textrm{e}^-}^{\,k}$:

7 $$\begin{aligned} i_\textrm{tot} = - 2F j_{\textrm{O}^{2-}}^{\,{\textrm{AO}}} - F j_{\textrm{e}^{-}}^{\,{\textrm{AO}}} = - 2F j_{\textrm{O}^{2-}}^{\,\textrm{BO}} - F j_{\textrm{e}^{-}}^{\,\textrm{BO}} \end{aligned}$$

The chemical potential of the component oxygen $\mu _{\textrm{O}_2}^k$ in both oxide phases consists of the electrochemical potentials of the oxide ions $\tilde{\mu }_{\textrm{O}^{2-}}^k$ and electronic charge carriers $\tilde{\mu }_{\textrm{e}^{-}}^k$:

8 $$\begin{aligned} \mu _{\textrm{O}_2}^k = 2 \, \tilde{\mu }_{\textrm{O}^{2-}}^k - 4 \, \tilde{\mu }_{\textrm{e}^{-}}^k \end{aligned}$$

Using Eq. (7) and (8) it is possible to rewrite Eq. (1) and (2), replacing the electrochemical potentials by the chemical potential of oxygen $\mu _{\textrm{O}_2}^k$ and the total current density $i_{\textrm{tot}}$:

9 $$\begin{aligned} -2F j_{\textrm{O}^{2-}}^{\,k}&= t_{\textrm{O}^{2-}}^k i_{\textrm{tot}} + \frac{t_{\textrm{e}^{-}}^k \sigma _{\textrm{O}^{2-}}^k}{4F} \frac{{\operatorname {d}\!{\mu }}_{\textrm{O}_2}^k}{{\operatorname {d}\!{x}}} \end{aligned}$$

10 $$\begin{aligned} -F j_{\textrm{e}^{-}}^{\,k}&= t_{\textrm{e}^{-}}^k i_{\textrm{tot}} - \frac{t_{\textrm{O}^{2-}}^k \sigma _{\textrm{e}^{-}}^k}{4F} \frac{{\operatorname {d}\!{\mu }}_{\textrm{O}_2}^k}{{\operatorname {d}\!{x}}} \end{aligned}$$

where $t_{\textrm{O}^{2-}}^k$ and $t_{\textrm{e}^{-}}^k$ are transference number of oxide ions and electronic charge carriers.^2^ Thus, in the absence of chemical potential gradients of oxygen ${\operatorname {d}\!{\mu }}_{\textrm{O}_2}^k / {\operatorname {d}\!{x}}$, the ratio between the electric charge transported by oxide ions and electronic charge carriers is only determined by the transference numbers. Assuming that there are no running redox reactions in the system, we can imply additionally to the conservation of (total) charge that the divergences ${\operatorname {d}\!{j}}_{\textrm{O}^{2-}}^{\,k} / {\operatorname {d}\!{x}}$ and ${\operatorname {d}\!{j}}_{\textrm{e}^-}^{\,k} / {\operatorname {d}\!{x}}$ of both charge carrier fluxes in the bulk phases are zero and the conservation of both fluxes at the interface. The latter results to the boundary conditions:

12 $$\begin{aligned} j_{\textrm{O}^{2-}}^{\,{\textrm{AO}}} = j_{\textrm{O}^{2-}}^{\,{\textrm{BO}}} \end{aligned}$$

13 $$\begin{aligned} j_{\textrm{e}^{-}}^{\,\textrm{AO}} = j_{\textrm{e}^{-}}^{\,\textrm{BO}} \end{aligned}$$

These conditions can only be fulfilled, if chemical potential gradients of oxygen ${\operatorname {d}\!{\mu _{\textrm{O}_2}^k}} / {\operatorname {d}\!{x}}$ are built up in the adjacent phases as additional driving forces altering the fluxes, see Fig. 4 b). In the depicted situation with an $i_\textrm{tot}$ pointing to the right and an oxide ion transference number of the AO phase smaller than that of the BO phase, a positive ${\operatorname {d}\!{\mu _{\textrm{O}_2}^\textrm{AO}}} / {\operatorname {d}\!{x}}$ in the AO phase and a negative ${\operatorname {d}\!{\mu _{\textrm{O}_2}^\textrm{BO}}} / {\operatorname {d}\!{x}}$ in the BO phase would increase the $j_{\textrm{O}^{2-}}^{\,\textrm{AO}}$ in the AO phase and decrease $j_{\textrm{O}^{2-}}^{\,\textrm{BO}}$ in the BO phase and *vice versa* for the electronic fluxes $j_{\textrm{e}^{-}}^{\,k}$. Thus, the boundary conditions are fulfilled if the chemical potential of oxygen $\Delta \mu _{\textrm{O}_2}^\textrm{int}$ is increased in the interface. If the current direction is reversed, the gradients are also reversed, resulting in a decrease of the chemical potential of oxygen at the boundary, see Fig. 5.

We assume composition independent conductivities $\sigma _{\textrm{O}^{2-}}^k$ and $\sigma _{\textrm{e}^{-}}^k$. If the divergences of both charge carrier fluxes are zero the chemical potential gradients ${\operatorname {d}\!{\tilde{\mu }_{\textrm{O}^{2-}}^k}}/{\operatorname {d}\!{x}}$, ${\operatorname {d}\!{\tilde{\mu }_{\textrm{e}^-}^k}}/{\operatorname {d}\!{x}}$ and ${\operatorname {d}\!{\mu }}_{\textrm{O}_2}^k / {\operatorname {d}\!{x}}$ are constant, *i.e.* the differential quotient is equal to the difference quotient:

14 $$\begin{aligned} \frac{{\operatorname {d}\!{\mu }}_{\textrm{O}_2}^k}{{\operatorname {d}\!{x}}} = \frac{\Delta \mu _{\textrm{O}_2}^k}{\Delta x^k} \end{aligned}$$

where $\Delta x^k$ are the extents of phase AO and BO in the electric current direction. The chemical potential $\Delta \mu _{\textrm{O}_2}^\textrm{int}$ of oxygen at the interface is given by:

15 $$\begin{aligned} \Delta \mu _{\textrm{O}_2}^\textrm{int} = \Delta \mu _{\textrm{O}_2}^\textrm{AO} = -\Delta \mu _{\textrm{O}_2}^\textrm{BO} \end{aligned}$$

The difference $\Delta \mu _{\textrm{O}_2}$ of the chemical potential of oxygen at the outer boundaries is set to zero and used as reference, see Fig. 5. Considering the condition in Eq. (12) to (15), the fluxes in Eq. (9) and (10), an expression for the oxygen potential difference $\Delta \mu _{\textrm{O}_2}^\textrm{int}$ of the interface to the outer boundaries can be derived, only dependent on the total electric current density $i_\textrm{tot}$:

16 $$\begin{aligned} \Delta \mu _{\textrm{O}_2}^\textrm{int}&= -4F \frac{t_{\textrm{O}^{2-}}^\textrm{AO} - t_{\textrm{O}^{2-}}^\textrm{BO}}{\frac{t_{\textrm{O}^{2-}}^\textrm{AO} \sigma _{\textrm{e}^{-}}^\textrm{AO}}{\Delta x^\textrm{AO}} + \frac{t_{\textrm{O}^{2-}}^\textrm{BO} \sigma _{\textrm{e}^{-}}^\textrm{BO}}{\Delta x^\textrm{BO}}} i_{\textrm{tot}} \nonumber \\&= 4F \frac{t_{\textrm{e}^{-}}^\textrm{AO} - t_{\textrm{e}^{-}}^\textrm{BO}}{\frac{t_{\textrm{O}^{2-}}^\textrm{AO} \sigma _{\textrm{e}^{-}}^\textrm{AO}}{\Delta x^\textrm{AO}} + \frac{t_{\textrm{O}^{2-}}^{\textrm{BO}} \sigma _{\textrm{e}^{-}}^\textrm{BO}}{\Delta x^\textrm{BO}}} i_\textrm{tot} \end{aligned}$$

## Footnotes

1 O$_2$ activity in air $a^0_{\textrm{O}_2} \approx$ 0.2 ($= p_{\textrm{O}_2}^0/p^0$, $p^0$ = 1 bar).
