# Supplementary material for: Strain release of substoichiometric (Zr,Y)O[image] phases formed by electrochemical reduction in single crystalline YSZ
Source: Sci Rep. 2026 Apr 11;16:12064. doi: 10.1038/s41598-026-45838-x (PMC13070037; doi:10.1038/s41598-026-45838-x)

1) Calibration of the nm-bar, Au nanoparticle

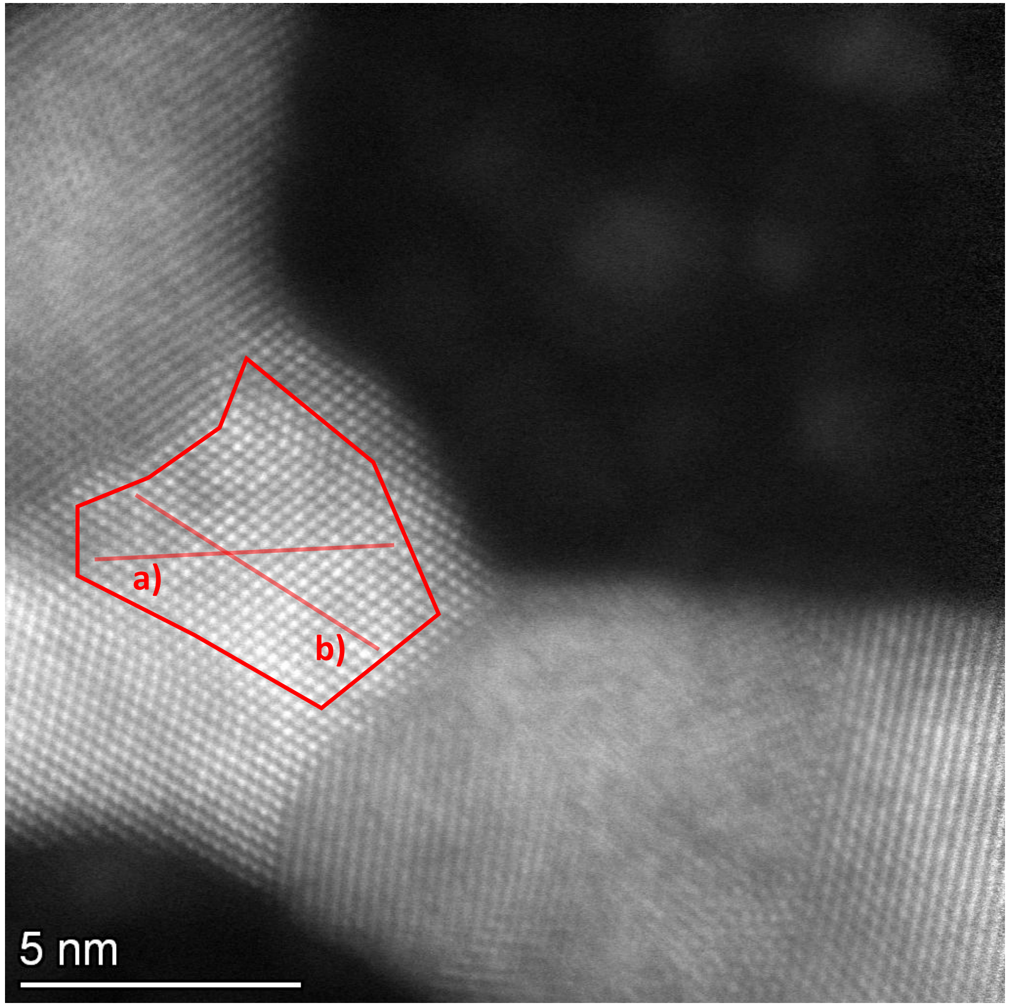

Gold nanoparticle,
STEM HAADF 5.60 Mx, 17.9 nm
1024 px x 1024 px

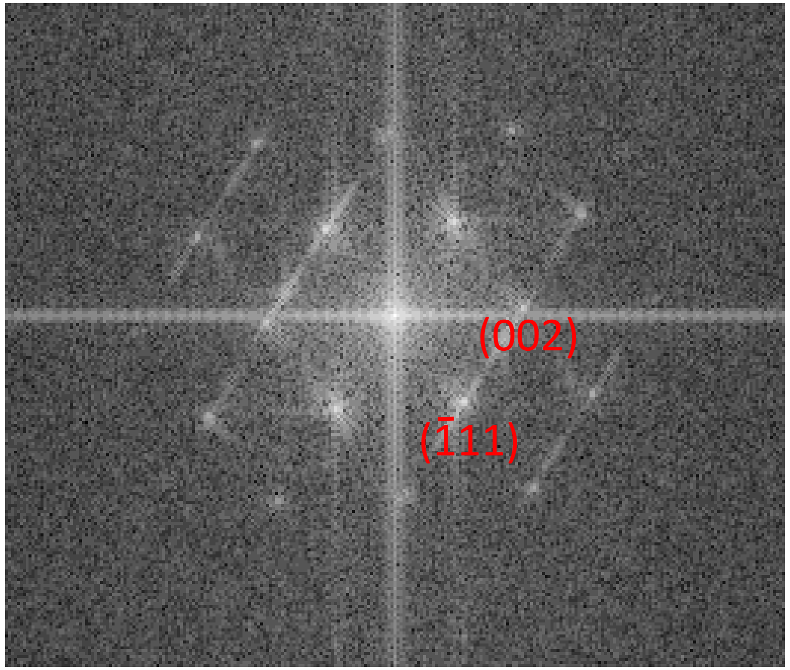

FFT of marked area

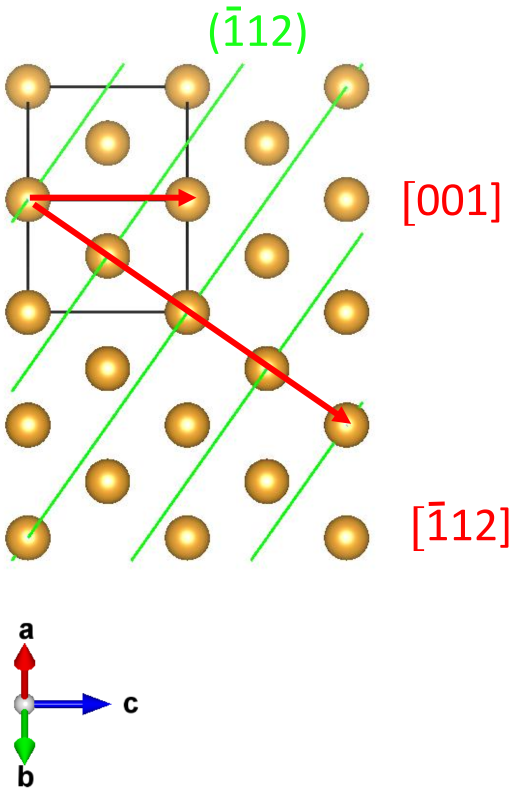

Crystallographic data from ICSD (release 2024.2), FIZ Karlsruhe, database entry no.: 163723

$d_{(001)}$ = 4.07090 Å

$d_{(112)}$ = 2.49291 Å

1) Calibration of the nm-bar, Au nanoparticle

Intensity profile a), along [001]

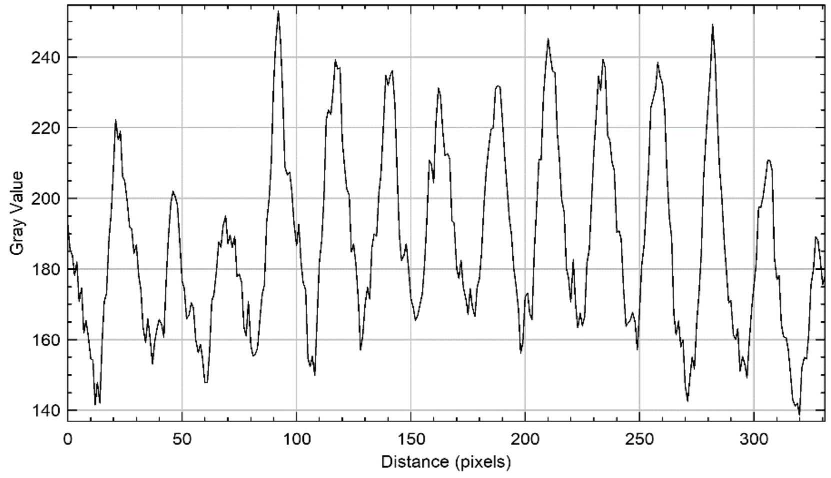

No. of atom (maximum) *vs.* distance

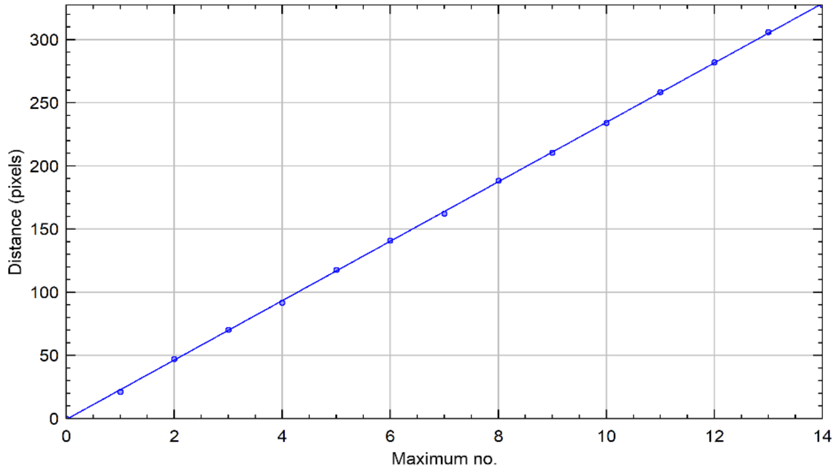

Slope = 23.52563 px/period

Scale = 23.52563 / 4.07090 px/Å
= **5.7787 px/Å**

Intensity profile b), along [$\bar{1}$12]

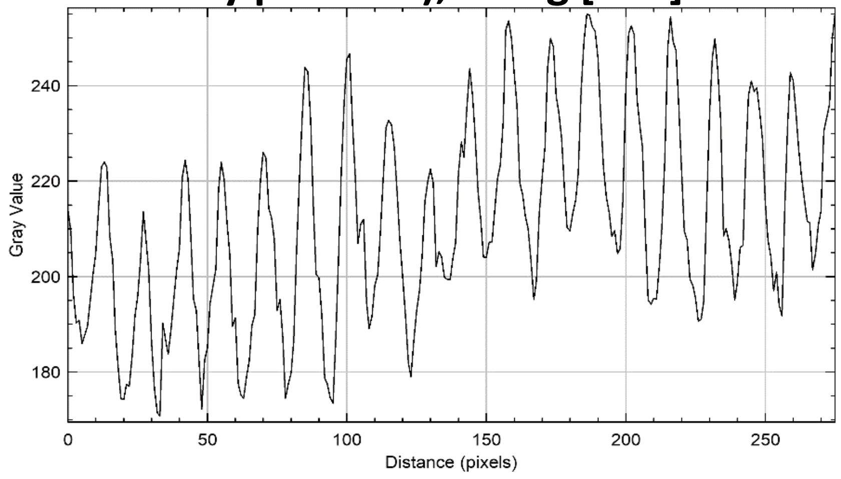

No. of atom (maximum) *vs.* distance

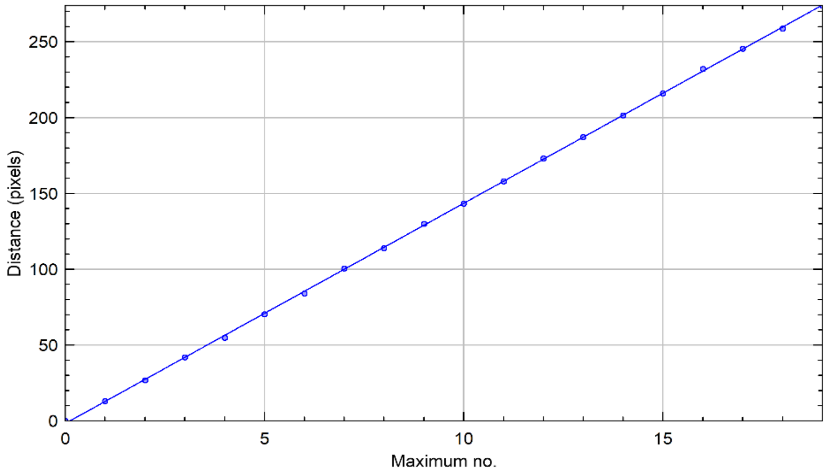

Slope = 14.52279 px/period

Scale = 14.52279 / 2.49291 px/Å
= **5.8256 px/Å**

Average:
Scale = (**5.80215 ± 0.022**) **px/Å**

1) Calibration of the nm-bar, Au nanoparticle

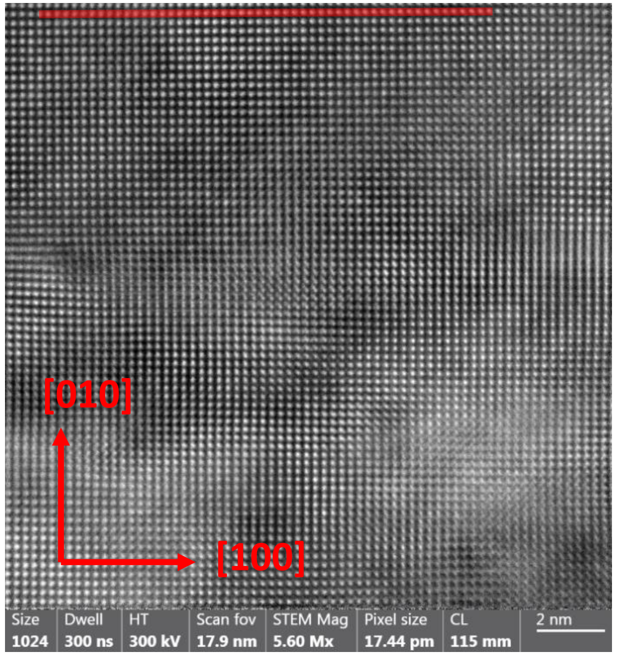

Heavily reduced zone at the cathode side,
STEM HAADF 5.60 Mx, 17.9 nm
1024 px x 1024 px

Intensity profile along [100], phase above „belt“

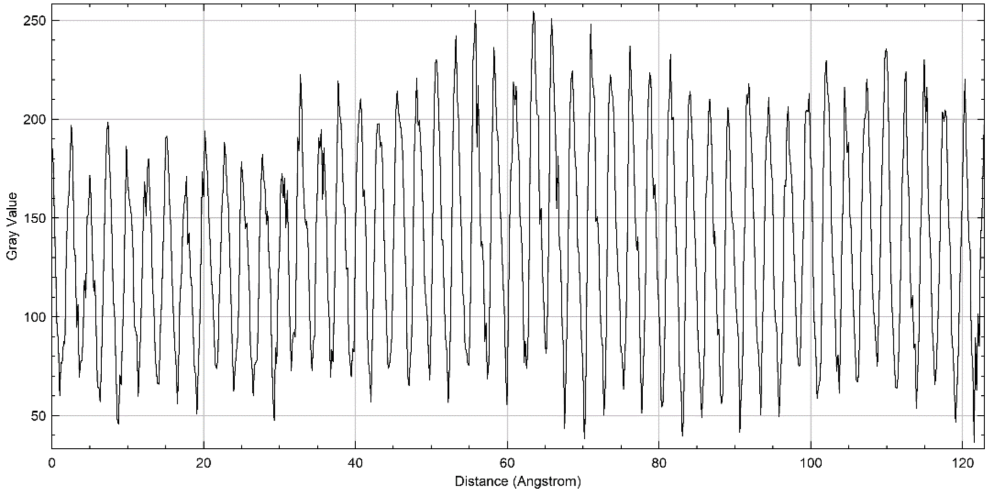

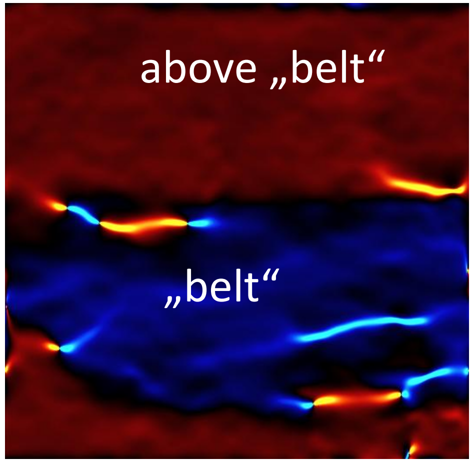

Strain tensor, ε_{yy}

No. of atom (maximum) *vs.* distance

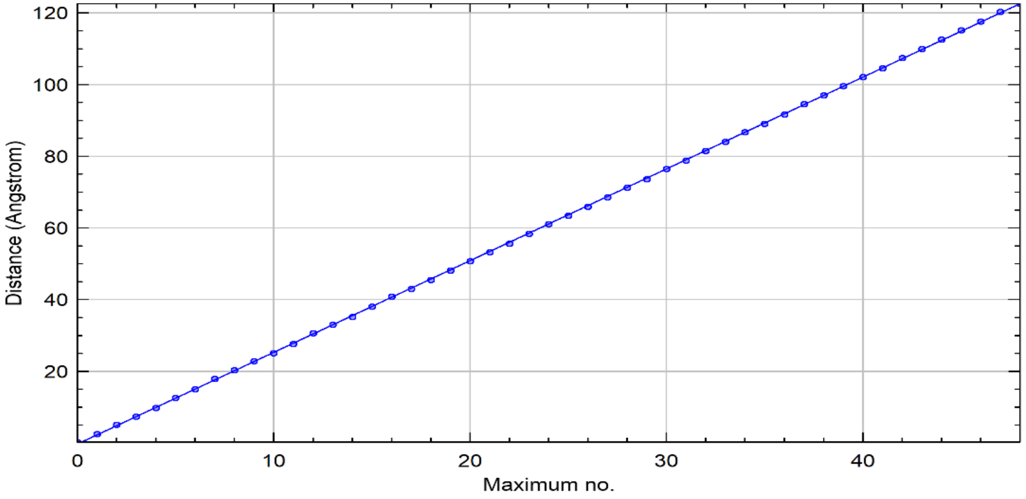

Slope = 2.55964 Å/period

$d_{(100)}$ = **5.1193 Å**

2) Re-calibration nm bar of micrograph #1 (Fig. 9.)

STEM/HAADF, 2048 px x 2048 px

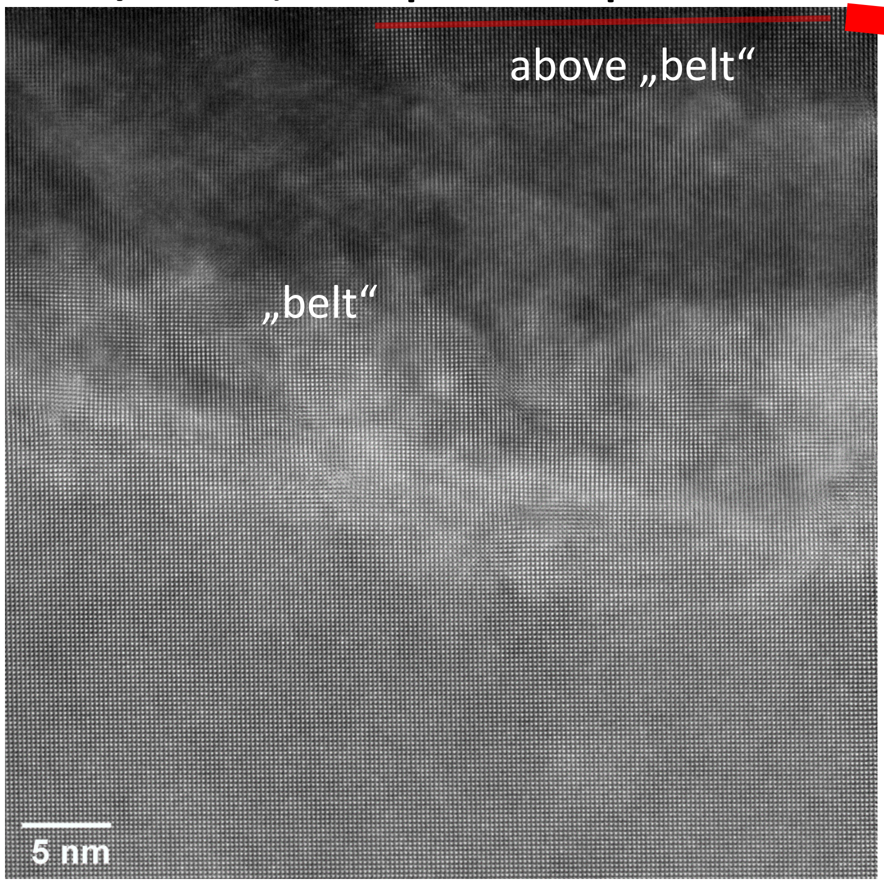

Intensity profile, along [001], phase above „belt“

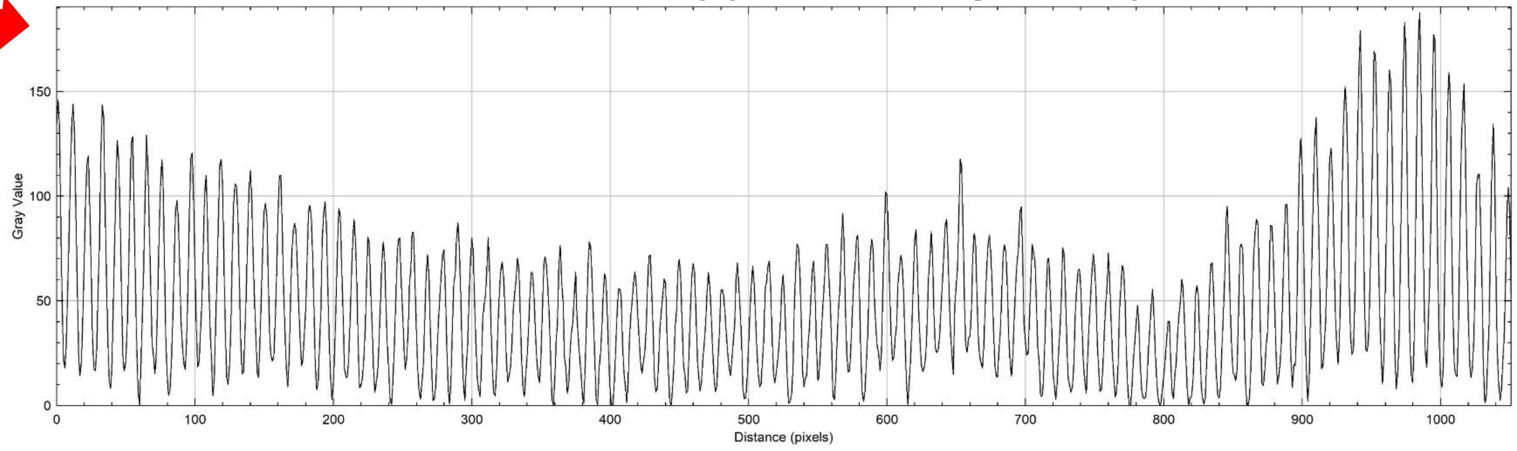

No. of atom (maximum) *vs.* distance

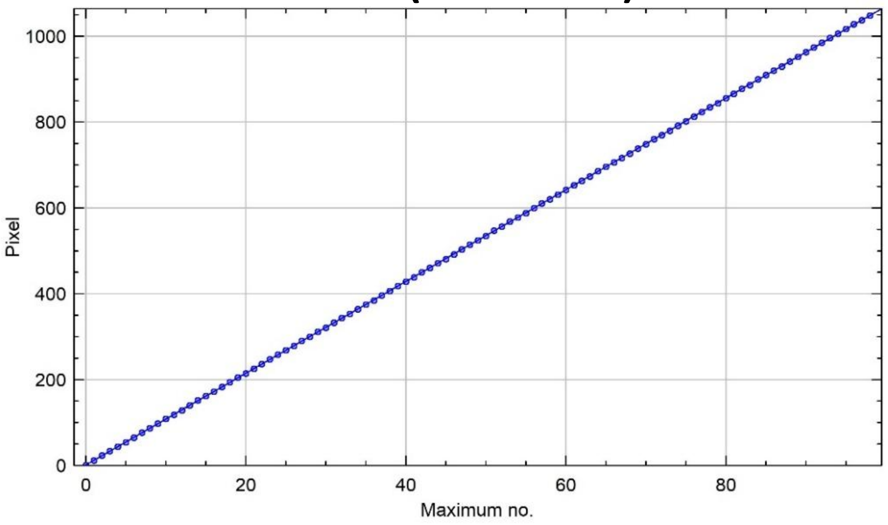

Slope = 10.68639 px/period

Result from page 3

2 x **10.68639 px — 5.1193 Å**

208.75 px — 50 Å (5 nm)

Scale = 4.1749 px/Å

3) Evaluation of lattice constants in micrograph #1 (Fig. 9.)

STEM/HAADF, 2048 px x 2048 px

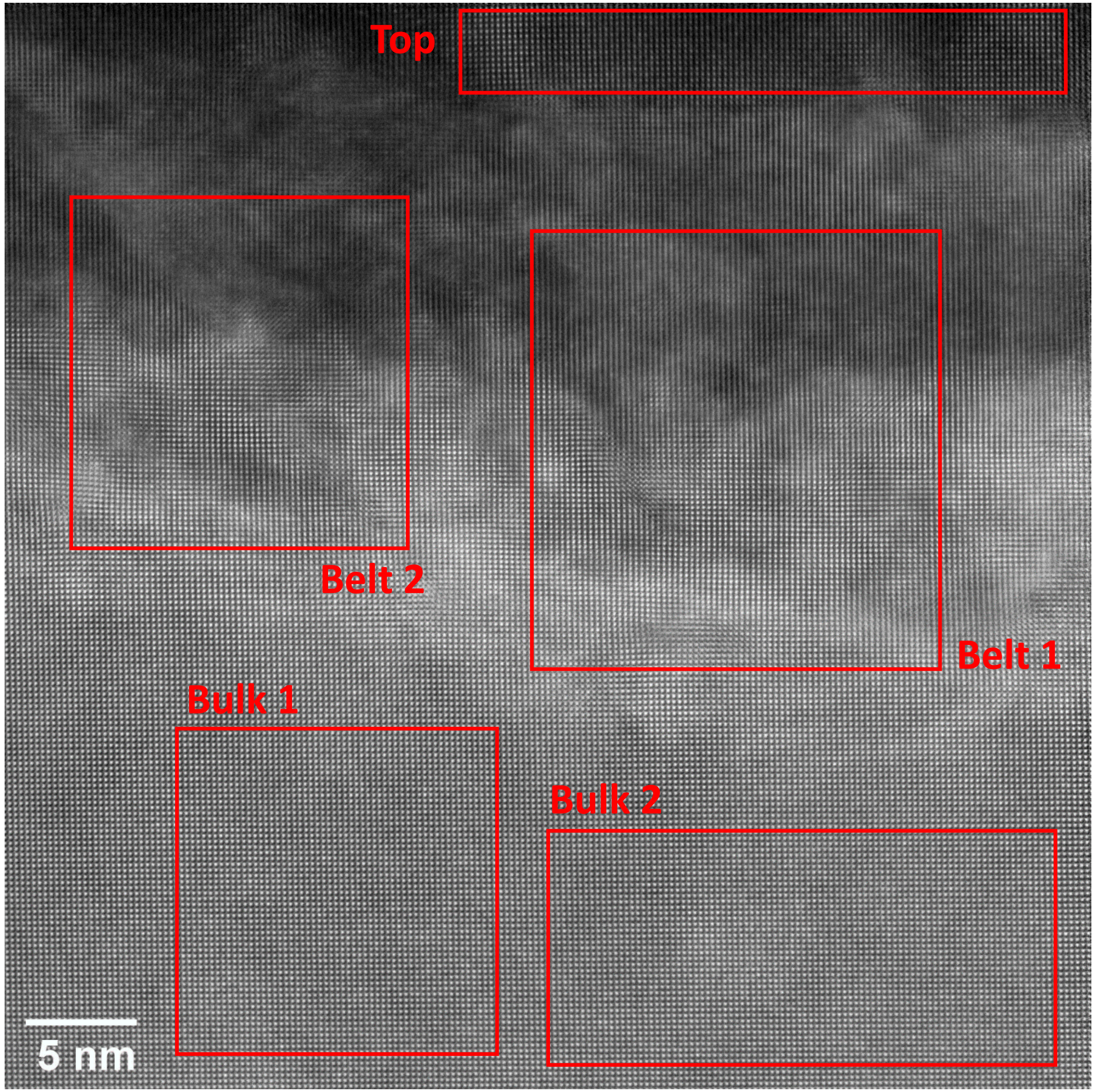

Heavily reduced zone at the cathode side

FFT, whole microslide

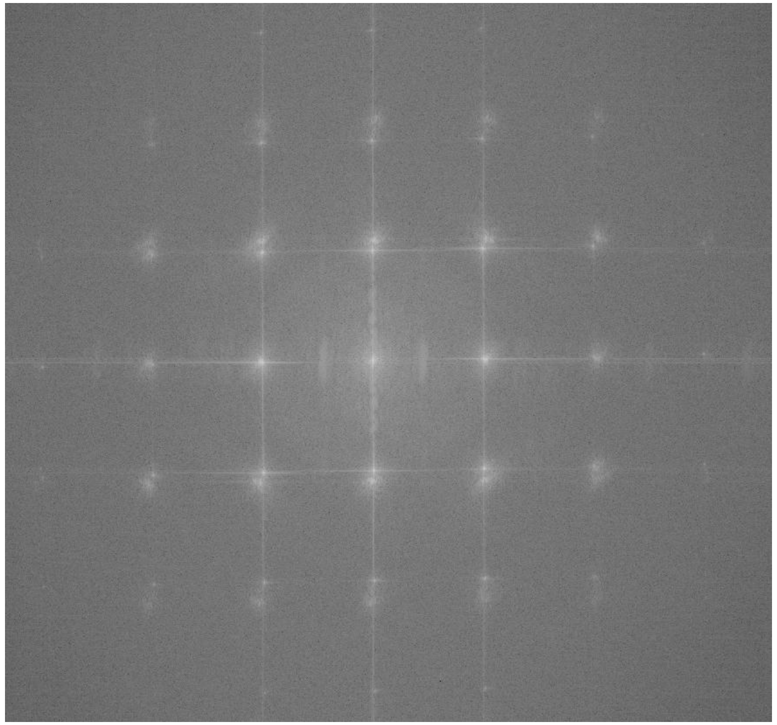

Scale = 4.1749 px/Å

Top

No.	g^{-1} / Å	ϑ / (°)	(hkl)	$d_{(100)}$, $d_{(010)}$ / Å
1	2.555	0.5	(200)	5.110
2	2.585	90.5	(020)	5.170
3	2.561	181.2	($\bar{2}$00)	5.122
4	2.561	270.5	(0$\bar{2}$0)	5.122

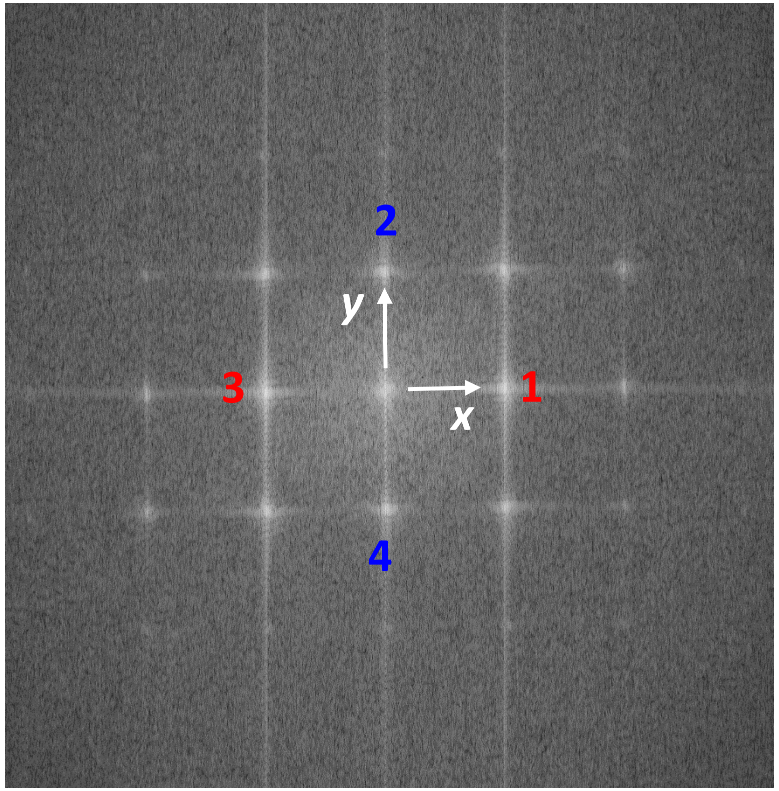

Bulk 1

No.	g^{-1} / Å	ϑ / (°)	(hkl)	$d_{(100)}$, $d_{(010)}$ / Å
1	2.562	0.4	(200)	5.124
2	2.589	90.3	(020)	5.178
3	2.563	181.7	($\bar{2}$00)	5.126
4	2.534	270.3	(0$\bar{2}$0)	5.068

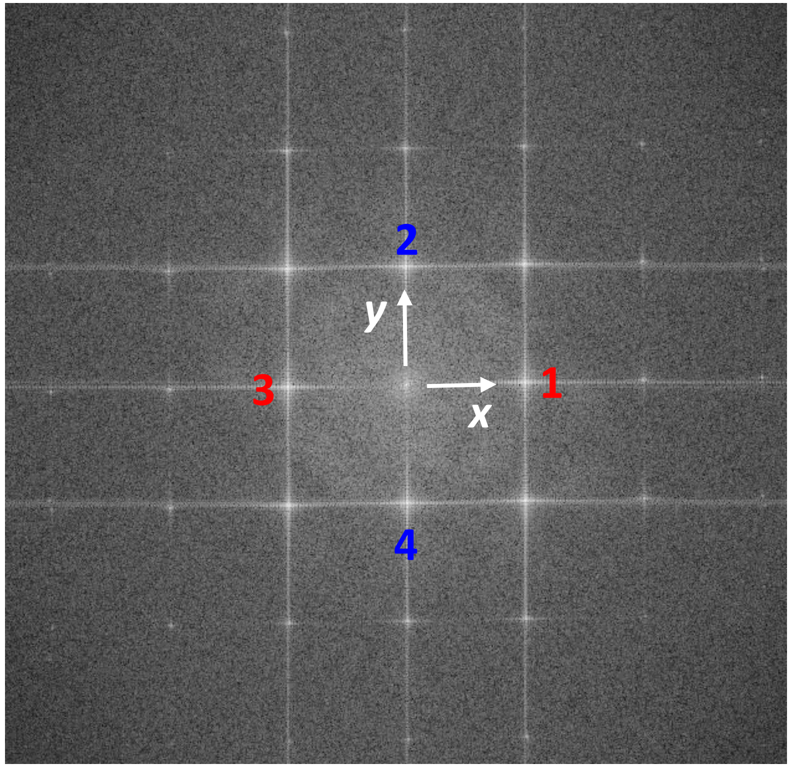

Bulk 2

No.	g^{-1} / Å	ϑ / (°)	(hkl)	$d_{(100)}$, $d_{(010)}$ / Å
1	2.561	0.7	(200)	5.122
2	2.575	90.2	(020)	5.150
3	2.571	181.4	($\bar{2}$00)	5.142
4	2.550	270.4	(0$\bar{2}$0)	5.100

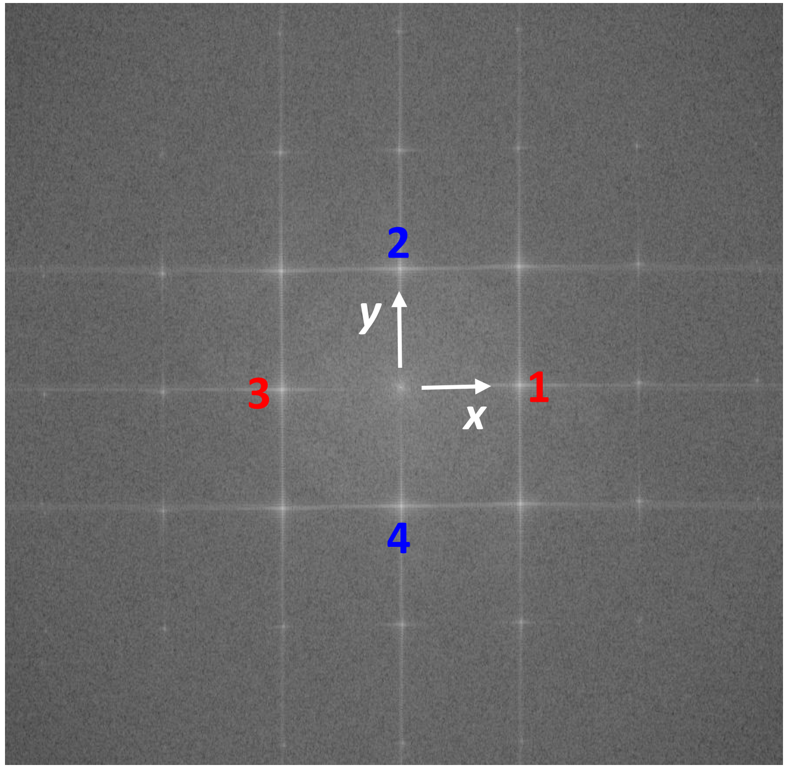

Belt 1

No.	g^{-1} / Å	ϑ / (°)	(hkl)	$d_{(100)}$, $d_{(010)}$ / Å
1	2.516	359.9	(200)	5.032
2	2.376	90.0	(020)	4.752
3	2.528	181.3	($\bar{2}$00)	5.056
4	2.311	269.9	(0$\bar{2}$0)	4.622

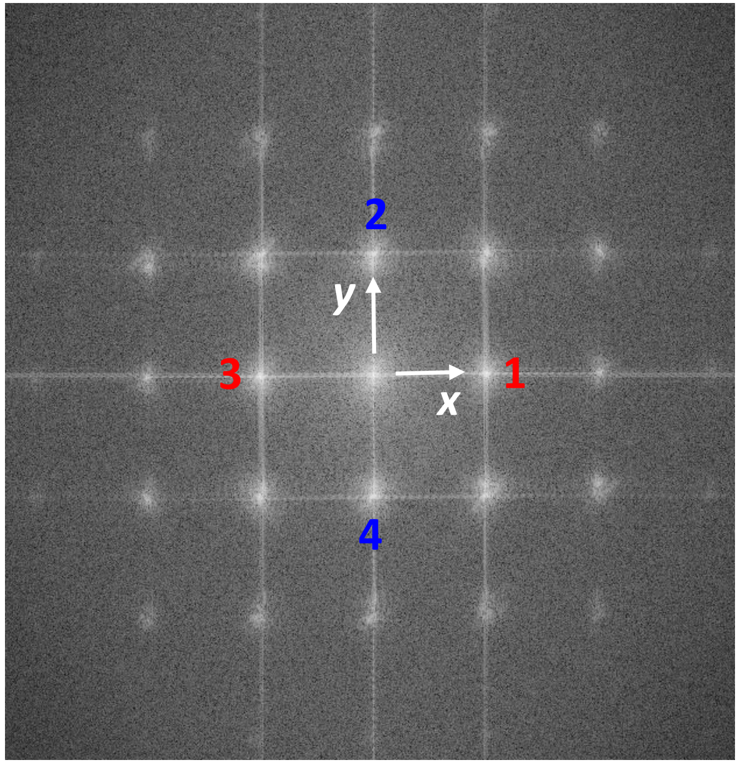

Belt 2

No.	g^{-1} / Å	ϑ / (°)	(hkl)	$d_{(100)}$, $d_{(010)}$ / Å
1	2.529	359.7	(200)	5.058
2	2.397	90.0	(020)	4.794
3	2.535	181.0	($\bar{2}$00)	5.070
4	2.330	270.0	(0$\bar{2}$0)	4.660

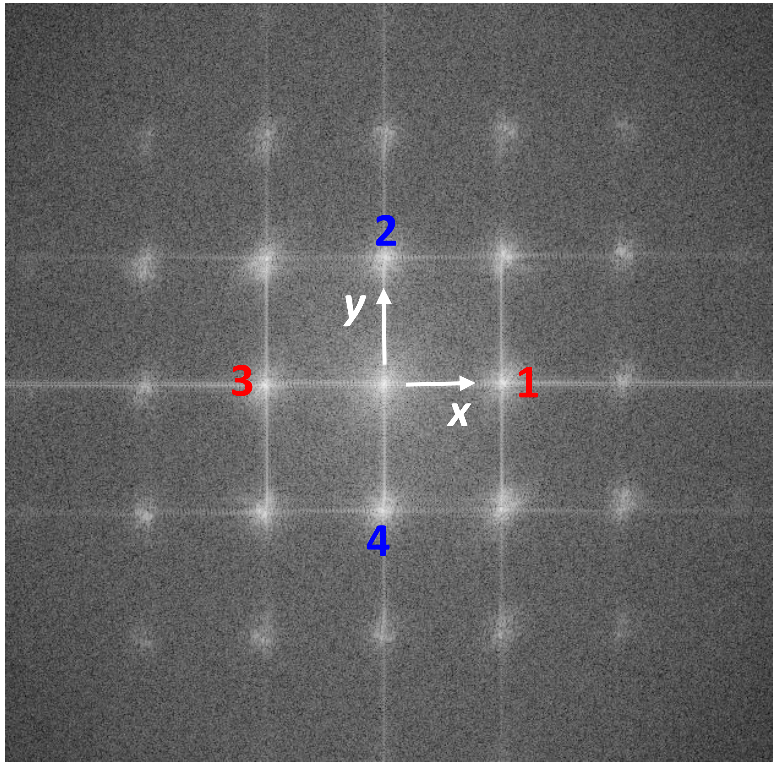

4) Summary, lattice constants in micrograph #1 (Fig. 9.)

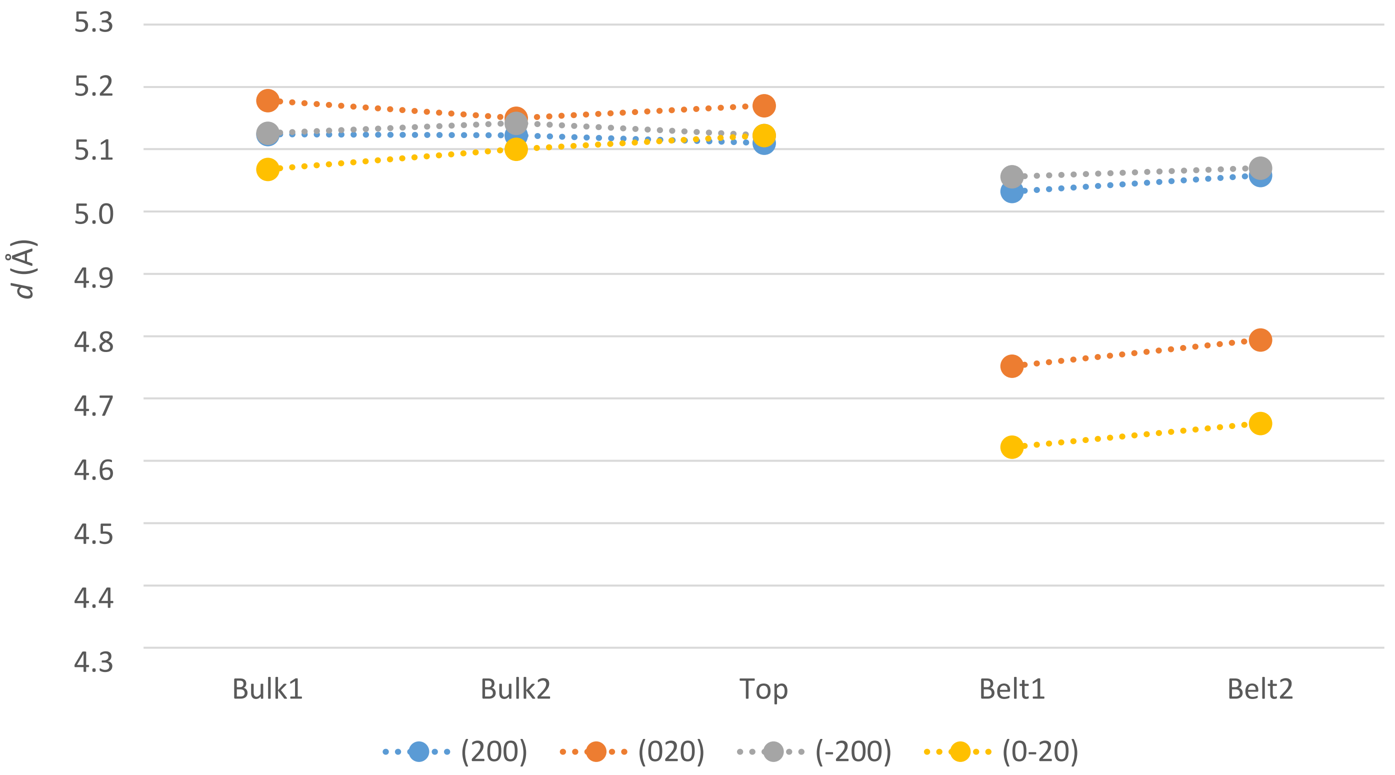

No significant differences between:

- the bulk region below („Bulk“) and above („Top“) the „belt-shaped“ zone
- (200), (020), $(\bar{2}00)$ and $(0\bar{2}0)$ of the bulk region below and above the „belt-shaped“ zone

Significant differences between:

- the bulk region below/above the „belt-shaped“ zone and the belt-shaped“ zone („Belt“)
- (200)/$(\bar{2}00)$ and (020)/$(0\bar{2}0)$ of the „belt-shaped“ zone

$\boldsymbol{d}_{(100)} = \boldsymbol{d}_{(010)}$ = (5.128 ± 0.009) Å

$\boldsymbol{d}_{(100)}$ = (5.054 ± 0.008) Å

$\boldsymbol{d}_{(010)}$ = (4.707 ± 0.040) Å

Error limits calculated according to $\sigma_{n-1}/\sqrt{n}$.

5) Lattice constant of YSZ vs. Y_2O_3 content

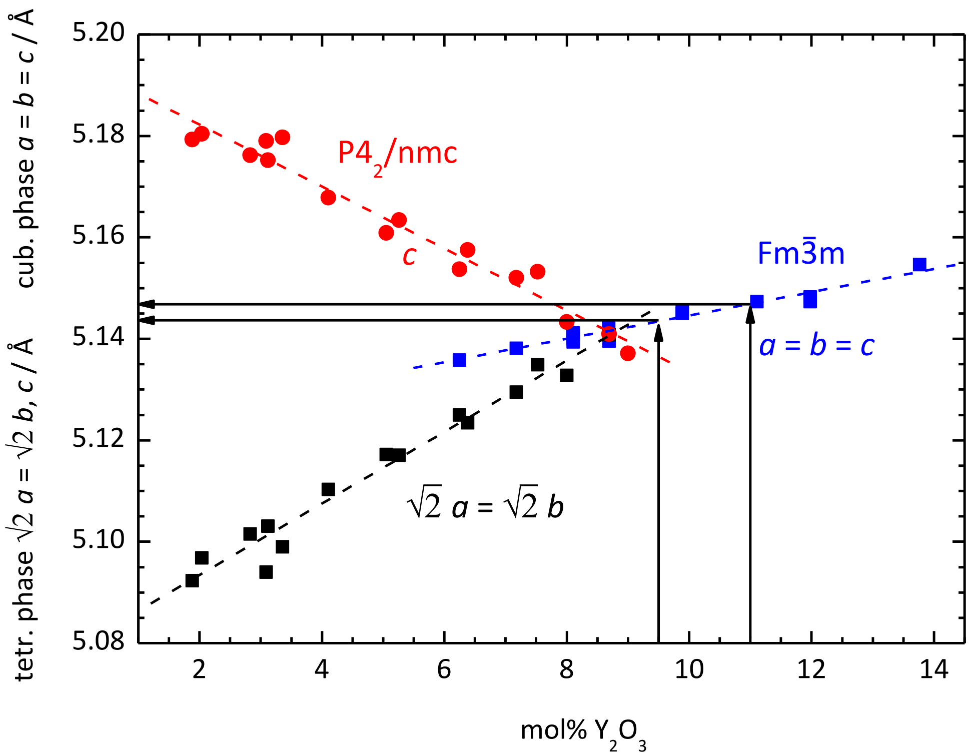

Crystallographic data from ICSD (release 2024.2), FIZ Karlsruhe, database entries no.:

60605, 19989, 165036, 75316, 181236, 238374, 238375, 238376, 238378, 238377, 238379, 238380, 280134, 75310, 75311, 75312, 75309, 165035, 90889, 90890, 79197, 86603, 89428, 655671

Krogstad et al., J. Am. Ceram. Soc. **94(12)**, 4548–4555, 2011
DOI: 10.1111/j.1551-2916.2011.04862.

$(1-x)\ ZrO_2 \cdot x\ Y_2O_3$

$$a = (5.1216\ \pm 0.0012)\ \text{Å} + (2.298 \pm 0.128\ ) \cdot 10^{-3} \frac{\text{Å}}{\text{mol\%}} \cdot\ x\ (\text{mol\%}\ Y_2O_3)$$

9.5YSZ: *a* = (5.143 ± 0.002) Å

11YSZ : *a* = (5.147 ± 0.003) Å

6) TEM/EDX analysis

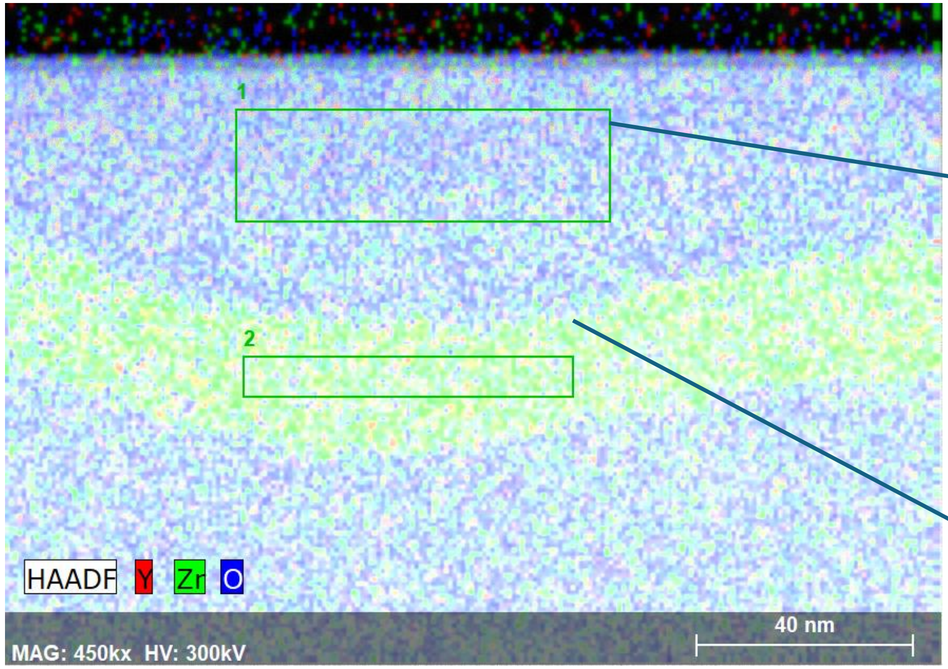

With the Cliff-Lorimer method

Results 1

Element	AN	series	Net	[wt.%]	[norm. wt.%]	[norm. at.%]	Error in wt.% (1 Sigma)
Zirconium	40	K-series	11942	73.4	73.4	52.9	2.3
Oxygen	8	K-series	6299	8.1	8.1	33.3	0.3
Yttrium	39	K-series	3564	18.5	18.5	13.7	0.7
			Sum:	100.0	100.0	100.0	

Results 2

Element	AN	series	Net	[wt.%]	[norm. wt.%]	[norm. at.%]	Error in wt.% (1 Sigma)
Zirconium	40.0	K-series	5176.0	79.3	79.3	72.2	2.7
Yttrium	39.0	K-series	1440.0	18.7	18.7	17.4	0.8
Oxygen	8.0	K-series	623.0	2.0	2.0	10.4	0.1
			Sum:	100.0	100.0	100.0	

Av. spectra

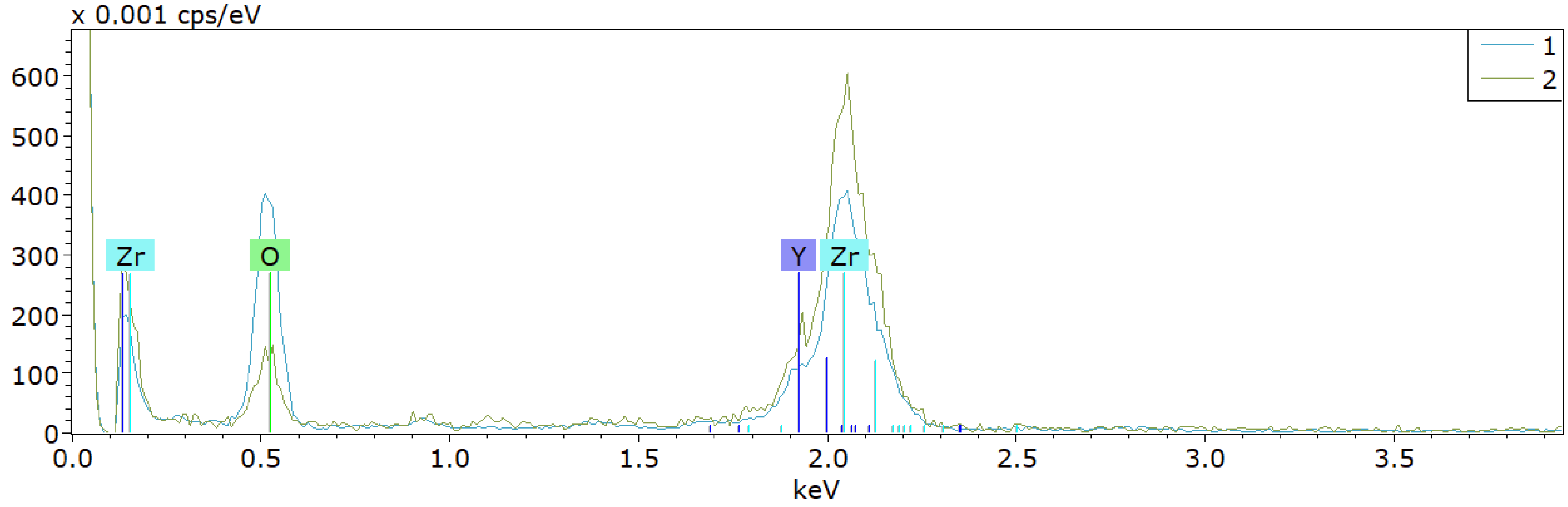

Supplement: Supplementary file 1 — Supplementary Information. [file 41598_2026_45838_MOESM1_ESM.pdf]
